# Identification of protein secretion systems and type III effectors in wood-associated bacteria of the genus *Xylophilus*

**DOI:** 10.1128/spectrum.00500-26

**Published:** 2026-07-06

**Authors:** Zoe Roux, Naama Wagner, Laurent Brottier, Tal Pupko, Ralf Koebnik

**Affiliations:** 1 PHIM, Université de Montpellier, Cirad, INRAE, Institut Agro, IRD 27037, Montpellier, France; 2 Shmunis School of Biomedicine and Cancer Research, George S. Wise Faculty of Life Sciences, Tel Aviv University 26745, Tel Aviv, Israel; Institute of Microbiology, Chinese Academy of Sciences, Beijing, China; University of Exeter, Exeter, United Kingdom; South China Agricultural University, Guangzhou, China

**Keywords:** protein secretion, type III effector, host-pathogen interactions, plant pathogens, *Xylophilus*, grapevine, comparative genomics, taxonomy

## Abstract

To gain insights into the biology of bacteria of the genus *Xylophilus*, including the grapevine pathogen *Xylophilus ampelinus*, we scrutinized all available genome sequences of the genus for the presence of type III secretion and flagellar systems. We found three distinct flagellar systems within the genus, one of which was present in all 12 strains for which good-quality genome sequences were available. The other two flagellar systems were only detected in one or two strains. We also identified two sub-classes of type III secretion systems, likely under control of the AraC-type transcriptional activator HrpX. One system with resemblance to systems from plant-pathogenic bacteria was only found in the grapevine pathogen. The other system was found in three strains of *Xylophilus*, all isolated from plant material. We predicted which genes are co-regulated with the type III secretion systems, as supported by the presence of strongly conserved HrpX-binding promoter elements. We identified about 40 type III effectors in the grapevine pathogen with homologs in plant pathogenic bacteria. In contrast, a rhododendron flower isolate had only two type III effector gene candidates with conserved HrpX-binding promoter elements, but many genes without homologs beyond the species. Finally, we predicted and confirmed three novel effector candidates from *X. ampelinus* to contain a functional type III secretion signal using an AvrBs1 reporter approach. The presence of type III effectors suggests that effector-triggered immunity may exist in grapevine or non-host plants and that strategies targeting type III effectors for resistance engineering may contribute to suitable control measures.

**IMPORTANCE:**

*Xylophilus* species include poorly characterized plant-associated bacteria, among them the grapevine pathogen *Xylophilus ampelinus*, yet their molecular mechanisms of host interaction have remained largely unknown. By curating public genomes and performing a genus-wide comparative analysis, this study clarifies *Xylophilus* taxonomy, reveals the distribution and evolutionary history of flagellar and type III secretion systems (T3SS), and identifies two distinct Hrp2/Hrc2-class T3SS types within the genus. Importantly, we define the predicted effector repertoires associated with these systems and experimentally validate three previously unrecognized type III effectors from *X. ampelinus*. These findings provide the first comprehensive framework for understanding virulence and host interaction strategies in *Xylophilus*, offering molecular targets and genomic markers relevant to plant pathology, disease surveillance, and the management of bacterial grapevine diseases.

## INTRODUCTION

*Xylophilus* is an understudied bacterial genus within the order *Burkholderiales*, family *Comamonadaceae*. These bacteria are primarily known for their association with plants, particularly with woody tissues, which is reflected in the genus name (from Greek: *xýlon*—wood; philus—friend, loving) (https://lpsn.dsmz.de/, accessed on 8 July 2025) (1). For instance, the type strain of the genus, belonging to the species *Xylophilus ampelinus* (formerly known as *Xanthomonas ampelina*), has been isolated from a diseased grapevine plant (2, 3). Bacteria belonging to another proposed species, *Xylophilus rhododendri*, have been isolated from the flowers of a *Rhododendron* plant (4). Other *Xylophilus* bacteria without species designation have been identified in metagenomic samples from woody tissues, e.g., from almond and blueberry (5, 6).

*Xylophilus* bacteria are of particular interest to plant pathologists and viticulturists due to their potential to damage valuable crops. One of the most notable species in this genus is the pathogen *X. ampelinus*, causing bacterial necrosis in grapevines (*Vitis vinifera*). Symptoms include discoloration of young shoots, necrotic spots on leaves, reddish-brown streaks on shoots, cracks, cankers, wilting, and dieback (7). Disease severity depends on cultivar susceptibility and environmental conditions (8). The bacterium enters plants through wounds or natural openings and colonizes xylem vessels, forming biofilms. Later, also phloem and cambial tissues were found to be infected. The resulting disruption of water transport causes tissue decay (9).

Although *X. ampelinus* is relatively rare compared to other grapevine pathogens, its impact in the affected regions can be serious (7). It is listed as a quarantine pathogen in several countries due to its potential to spread and damage viticulture industries (10). Understanding and monitoring *X. ampelinus* is critical to maintaining healthy vineyards and ensuring the sustainability of grape production, particularly in Europe, where it has been of most concern in the past.

Many plant-associated bacteria of the phylum Pseudomonadota, including Alphaproteobacteria (e.g., *Bradyrhizobium*, *Mesorhizobium*, *Sinorhizobium*), Betaproteobacteria (e.g., *Acidovorax*, *Burkholderia*, *Paracidovorax*, *Papaburkholderia*, *Ralstonia*), and Gammaproteobacteria (e.g., *Pantoea*, *Pseudomonas*, *Xanthomonas*), rely on a specialized protein secretion system for their interaction with the plant, be it a beneficial or a pathogenic relationship (11–13). The type III secretion system (T3SS) injects effector proteins, called type III effectors (T3Es), into the host cells to the benefit of the bacteria, thereby favoring the colonization (infection or symbiotic) process. Historically, the T3SS was first characterized in human pathogens, focusing on species of *Yersinia*, *Salmonella*, and *Shigella* (14).

Based on sequence similarities, T3SSs have been classified into seven major classes, reflecting the bacteria from which they were first characterized (Chlamy, Hrp1/Hrc1, Hrp2/Hrc2, SPI-1 [Inv/Mxi/Spa], SPI-2 [Esc/Ssa], Rhizo [Rhc], Ysc) (15–17). The T3SS is a multi-component protein complex that contains 13 conserved proteins in different copies, namely the ATPase SctN, the sorting platform consisting of SctK, SctL, and SctQ, the inner membrane secretion apparatus consisting of SctR, SctS, SctT, SctU, and SctV, the IM ring consisting of SctD and SctJ, the inner rod protein SctI, the outer membrane secretin SctC, and the needle filament SctF (16), according to the unified nomenclature (18). Nine of them (SctJLNQRSTUV) have clear homologs in the flagellar basal body, and two (SctP and SctF) have functional and structural counterpart analogs in the flagellum. Compelling evidence suggests that the non-flagellar T3SS evolved from the flagellum (16, 19, 20). The two gate proteins SctU (and its flagellar homolog FlhB) and SctV (and its flagellar homolog FlhA) constitute two of the most conserved components of the T3SS (16, 21). As such, they are considered a signature for T3SS presence (or remnants thereof) and allow classification of the non-flagellar T3SS into seven classes (17, 22).

Different T3SS classes exhibit distinct regulatory mechanisms governing gene expression (23). For instance, the Hrp1/Hrc1 system is controlled by the extracytoplasmic function sigma factor HrpL, which is thought to direct the RNA polymerase to the “*hrp* box” in the promoters of T3SS genes (24). The *hrpL* gene itself is regulated by a heterohexamer of HrpR and HrpS in strains of *Pseudomonas syringae* or by an HrpS homohexamer in the Enterobacterales (24). The other plant-associated T3SS, Hrp2/Hrc2, is under the control of the two-component response regulator HrpG. It is thought that HrpG is post-translationally activated by protein phosphorylation by a hitherto unknown component, leading to transcriptional activation of the *hrpX* gene (25). The AraC-type regulator HrpX (in *Acidovorax* and *Xanthomonas*)/HrpB (in *Burkholderia* and *Ralstonia*) then binds to a conserved promoter element, the *hrpII*/plant-inducible promoter (PIP) box, thus regulating T3SS and T3E genes (26). Activation of HrpX-controlled genes strictly relies on the presence of a PIP box and a properly spaced –10 promoter motif (27–30). Many, if not most, HrpX/HrpB-regulated genes encode components of the T3SS machinery, T3Es, or secreted hydrolases, such as endoglucanases, proteases, esterases, and pectinases (30–36). The presence of HrpX-regulated promoters, identified either experimentally or *in silico*, is used to predict T3E genes, which can then be confirmed and characterized in more detail (29, 31, 35).

In order to better understand the host-pathogen molecular interactions within the genus *Xylophilus*, we aimed at predicting and comparing T3SSs and candidate T3E proteins at a genome-wide level. Our work provides new insights into the evolution of T3SSs in the genus *Xylophilus* and the presence of T3Es in these species. For three candidate T3Es, we provide evidence that they are bona fide effectors likely translocated by the T3SS and injected into grapevine plants.

## MATERIALS AND METHODS

### Biological material

#### Bacterial strains and growth conditions

Strains of *Escherichia coli* (DH10B) and *Xanthomonas campestris* (8000 and its mutants in the T3E *avrBs1* and the type III secretion gene *hrcV*) were used in this study (37–39). The *X. campestris* strains were cultivated at 28°C on solid peptone-sucrose-agar (PSA) medium (peptone 10 g/L, sucrose 10 g/L, agar 16 g/L, glutamic acid 1 g/L). *Escherichia coli* cells were grown at 37°C on lysogenic broth (LB) agar plates (LB Lennox agar 35 g/L; Euromedex, Souffelweyersheim, France) or in liquid LB medium (tryptone 10 g/L, yeast extract 5 g/L, NaCl 10 g/L). Antibiotics were added, when needed, to the media at the following final concentrations: 100 mg/L for rifampicin and 5 mg/L for tetracycline.

#### Plasmids

The plasmid pBBR1MCS-3::avrBs1_59-445_ is a derivative of the plasmid pBBR1MCS-3 (40). It encodes an N-terminally truncated variant of AvrBs1 from *X. campestris*. This variant lacks its own type III secretion signal and is used to construct translational reporter gene fusions with presumed type III secretion signals from candidate T3E genes (39). Reporter fusions were constructed by GenScript (Piscataway Township, NJ, USA) from synthetic DNA that consisted of the 100-base-pair noncoding sequence upstream of the translation initiation codon, as well as the 300-base-pair coding sequence that would include the N-terminal type III secretion signal from the candidate genes. The helper plasmid pRK2013 was used to transfer plasmids via conjugation from *E. coli* to *Xanthomonas* by triparental mating (41).

#### Plant material

The pepper cultivar ECW-10R, which triggers a hypersensitive response upon perception of AvrBs1 by the corresponding *Bs1* resistance gene product, was used for plant inoculation experiments (42). Plants were grown in a greenhouse with natural daylight cycles at 27°C ± 5°C and 60% relative humidity.

### Bioinformatic analyses

#### Genomic resources

Genomic resources, such as genome sequences, genome annotations, predicted open reading frames (ORFs), and proteomes, were retrieved from the National Center for Biotechnology Information (NCBI) data set resource (https://www.ncbi.nlm.nih.gov/datasets/) (43). Quality and correct taxonomic classifications were evaluated using the Type (Strain) Genome Server (TYGS; https://tygs.dsmz.de/), the Microbial Genomes Atlas (MiGA; https://gateway.microbial-genomes.org/), and the ContEst16S algorithm (http://www.ezbiocloud.net/tools/contest16s) (44–46). Genome-wide average nucleotide identities (ANI) were calculated using FastANI version 1.3 (47), as implemented at the French Galaxy server (https://usegalaxy.fr/) (48).

#### Sequence comparisons and phylogenetic trees

Sequence similarity searches and pairwise sequence comparisons were performed at the NCBI using the BLAST and Needleman-Wunsch algorithms, respectively (43, 49, 50). Multiple sequence alignments were generated with MUSCLE (51), as implemented at the EMBL’s European Bioinformatics Institute (52). To compare T3SS gene clusters, the corresponding regions were extracted from the annotated GenBank files using the Artemis genome browser (53). Clinker was used for the comparison and visualization of genomic regions (54).

Phylogenetic trees based on sequence comparisons were generated at Phylogeny.fr (https://www.phylogeny.fr/), with default parameters (55). Core genome-wide comparisons were performed using M1CR0B1AL1Z3R 2.0 (https://microbializer.tau.ac.il/) (56), and phylogenetic trees were inferred with bootstrap support values, with DSM 13620 used as the outgroup, and all other parameters set to their default values. For better visualization of the trees, we used the Interactive Tree of Life tool, v6 (57).

#### Prediction of protein secretion systems and type III effectors

Protein secretion systems were predicted using TXSScan, as implemented in the Galaxy platform at the Institut Pasteur (https://galaxy.pasteur.fr/root?tool_id%20=%20toolshed.pasteur.fr/repos/odoppelt/txsscan/TXSScan/1.0.2) (58). The type IV and type VI secretion systems were also predicted using SeCreT4 and SeCreT6 (https://bioinfo-mml.sjtu.edu.cn/SecReT4/; https://bioinfo-mml.sjtu.edu.cn/SecReT6/), respectively (59, 60).

To predict T3Es, we employed Effectidor II, a pan-genomic AI-based algorithm for the prediction of T3Es (https://effectidor.tau.ac.il) (61). The input consisted of predicted ORFs and GFF3 annotations, as available from NCBI, targeting the genomes of five strains that harbor an Hrp2/Hrc2-related T3SS (assembly accession numbers GCA_003217575.1, GCA_024832295.1, GCA_033546935.1, GCA_009906855.1, and GCA_026951875.1 for strains CECT7646, CFBP 1192, GOD-11R, KACC 21265, and Kf1, respectively). The predicted proteomes of strains ASV27, bin23, GW821-FHT01B05, Leaf220, and SP210_2 were used as a negative training set (assembly accession numbers GCF_016428875.1, GCA_036946225.1, GCA_038961845.1, GCA_001421705.1, and GCF_913776965.1, respectively) to represent strains that do not encode a Hrp2/Hrc2-related T3SS. Search options included prediction of the type III secretion signal and searching for the presence of a PIP-box motif in the upstream region of all ORFs. All other parameters were left at their default settings. We included all predictions with scores higher than 0.25. This cutoff was selected because candidates with scores near this threshold showed sequence similarity to known effectors, whereas candidates below this threshold did not.

#### Other bioinformatic tools

Protein domains and sequence motifs were predicted using InterProScan (https://www.ebi.ac.uk/interpro/) (62). Three-dimensional structures of proteins were predicted by AlphaFold3 (https://alphafoldserver.com/welcome) (63). FoldSeek was used to identify structural homologs (https://search.foldseek.com/) (64). To reduce the complexity of data sets, groups of homologous sequences were clustered using CD-Hit at usegalaxy.fr (Galaxy Version 4.8.1) (65). Signal sequences for secretion via the Sec or Tat pathway were predicted with SignalP 6.0 (https://services.healthtech.dtu.dk/services/SignalP-6.0/) (66). GeNomad in its Galaxy version 1.11.1 was used to identify mobile genetic elements, such as prophages (67). Genome sequences were scanned for perfect PIP patterns in the Artemis genome browser, using TTCGB-N_15_-TTCGB-N_30-32_-YANNNT as a query.

All Galaxy workflows are provided at https://doi.org/10.5281/zenodo.19733922.

### Plant inoculation experiments

Eight- to twelve-week-old plants of the pepper cultivar ECW-10R were used for plant inoculation experiments (42). Bacteria were grown on PSA medium, resuspended in 10 mM MgCl_2_, and diluted to an optical density at 600 nm wavelength of 0.1. A needleless 1-mL plastic syringe was used to inoculate the bacterial solution from the lower side of the pepper leaf into the apoplast. Each bacterial solution was inoculated on at least two different leaves from at least two different plants. Experiments were performed at least twice. Plant reactions were scored over a period of 7 days after the inoculation.

## RESULTS

### Clarification of taxonomic status of publicly available genome sequences

In order to obtain an overview of the presence of virulence-associated secretion systems in the genus *Xylophilus*, we first extracted all genome sequences that are assigned to this bacterial genus from NCBI (https://www.ncbi.nlm.nih.gov/datasets/genome/). NCBI GenBank lists 19 genome sequences for this genus (accessed on 29 July 2025), 6 for the species *X. ampelinus*, 1 for the species *X. rhododendri*, and 12 without species assignment (Table 1).

**TABLE 1:** *Xylophilus* genome sequences at NCBI GenBank^*a*^

Organism name at NCBI	Strain/isolate	GenBank assembly	RefSeq assembly	Genome accession number	Number of contigs	Size (bp)	BioProject	BioSample	Reference
*Xylophilus ampelinus*	CECT7646^T^	GCA_003217575.1	GCF_003217575.1	QJTC01	66	3,731,505	PRJNA463320	SAMN09074800	
*Xylophilus ampelinus*	CFBP 1192^T^	GCA_024832295.1	GCF_024832295.1	JAMOFZ01	85	3,736,570	PRJNA843606	SAMN28766069	Portier et al. (68)
*Xylophilus ampelinus*	Xylophilus_ampelinus_ BgEED09^*b*^	GCA_901875635.1	GCF_901875635.1 ^*c*^	CABFMN01	161	6,174,221	PRJEB32184	SAMEA5664384	
*Xylophilus ampelinus*	bin23	GCA_036946225.1		JAQIJO01	14	3,596,058	PRJNA922445	SAMN32653142	
*Xylophilus ampelinus*	CCH5-B3^*b*^	GCA_001556675.1	GCF_001556675.1 ^*c*^	LSJH01	1,406	6,019,991	PRJNA299404	SAMN04299458	Soto-Giron et al. (69)
*Xylophilus ampelinus*	ERR594292_binner13_ Refined_23^*b*^	GCA_964011595.1		CAXGPN01	932	5,771,620	PRJEB51168	SAMEA115421838	
*Xylophilus rhododendri*	KACC 21265^T^	GCA_009906855.1	GCF_009906855.1	CP047650.1, CP047651.1	2	5,873,400	PRJNA600143	SAMN13783577	Lee et al. (4)
*Xylophilus* sp.	ASV27	GCA_016428875.1	GCF_016428875.1 ^*c*^	JAELTO01	469	4,734,944	PRJNA682646	SAMN17004937	Goyal et al. (70)
*Xylophilus* sp.	cluster_DBSCAN_ round5_1	GCA_009914555.1	GCF_009914555.1	WNDY01	129	4,706,822	PRJNA531449	SAMN12995593	Waterworth et al. (71)
*Xylophilus* sp.	GOD-11R	GCA_033546935.1	GCF_033546935.1	CP137854.1	1	5,127,500	PRJNA1034763	SAMN38071784	
*Xylophilus* sp.	Go_Prim_bin_55	GCA_017990095.1		JAGNON01	437	2,320,559	PRJNA524094	SAMN18119707	Schneider et al. (72)
*Xylophilus* sp.	Gw_Inlet_bin_57	GCA_018006615.1		JAGMXZ01	431	1,897,017	PRJNA524094	SAMN18119261	Schneider et al. (72)
*Xylophilus* sp.	Gw_Prim_bin_50	GCA_018005875.1		JAGMZJ01	323	1,282,324	PRJNA524094	SAMN18119294	Schneider et al. (72)
*Xylophilus* sp.	Gw_UH_bin_252	GCA_017989255.1		JAGNQF01	338	1,400,660	PRJNA524094	SAMN18119505	Schneider et al. (72)
*Xylophilus* sp.	GW821-FHT01B05^*b*^	GCA_038961845.1	GCF_038961845.1 ^*c*^	CP152408.1	1	5,832,500	PRJNA1101085	SAMN40985315	Biggs et al. (73)
*Xylophilus* sp.	Kf1^*b*^	GCA_026951875.1		WPBX01	1,939	5,678,042	PRJNA593051	SAMN13451138	
*Xylophilus* sp.	Leaf220	GCA_001421705.1	GCF_001421705.1	LMKO01	28	4,483,623	PRJNA297956	SAMN04151686	Bai et al. (74)
uncultured *Xylophilus* sp.	SP51_3_metabat2_ genome_mining.45	GCA_913777525.1		CAJYSJ01	494	3,038,960	PRJEB45634	SAMEA8944104	Su et al. (75)
uncultured *Xylophilus* sp.	SP210_2_metabat2_ genome_mining.41	GCA_913776965.1	GCF_913776965.1	CAJYQL01	136	4,051,675	PRJEB45634	SAMEA8944525	Su et al. (75)
*Xenophilus aerolatus*	DSM 19424^T*b*^	GCA_029968055.1		JASCNX01	11	5,117,920	PRJNA967166	SAMN34578380	Kim et al. (76)
*Xenophilus arseniciresistens*	YW8^T^	GCA_028023875.1	GCF_028023875.1	JAQIPB01	27	5,017,807	PRJNA922719	SAMN32673052	Li et al. (77)
*Xenophilus azovorans*	DSM 13620^T^	GCA_000745855.1	GCF_000745855.1	JQKD01	137	6,529,337	PRJNA221063	SAMN02745804	Blümel et al. (78)

^*a*^ Strain isolates Xylophilus_ampelinus_BgEED09, CCH5-B3, and ERR594292_binner13_Refined_23 do not belong to the genus *Xylophilus*. Sample bin23 appears to belong to the genus *Xylophilus* but not to the species *X. ampelinus*. Type strains are indicated by a superscript T.

^*b*^ Warning at NCBI: contaminated.

^*c*^ RefSeq assembly suppressed.

The Type strain Genome Server (TYGS; https://tygs.dsmz.de) was applied to confirm the taxonomic classification (accessed on 30 July 2025) (Table S1a) (1). Only the two sequences of strains CECT7646 and CFBP 1192, both corresponding to the same type strain, which was isolated from a necrotic grapevine plant in Crete, Greece, in 1966, were reported to belong to the species *X. ampelinus*. Strain KACC 21265 was confirmed to belong to the species *X. rhododendri*. Eight genome sequences (strains/isolates ASV27, bin23, Cluster_DBSCAN, GOD-11R, GW821-FHT01B05, Leaf220, SP51_3, SP210_2) were proposed as potential new species of the genus *Xylophilus*. Three of the genome sequences (strains/isolates BgEED, CCH5-B3, ERR594292) were proposed as potential new species of the genus *Xenophilus*. Four surprisingly small genome sequences, originating from metagenome assemblies from wastewater treatment plants in Germany (72), were proposed as potential new species of the genera *Acidovorax* (Go_Prim_bin_55, Gw_Prim_bin_50, Gw_UH_bin_252) or *Macromonas* (Gw_Inlet_bin_57). Lastly, the genome sequence of strain Kf1 was flagged by TYGS as a potentially unreliable identification result due to potential sequence contamination, belonging to the species *Staphylococcus epidermidis*. This observation was confirmed by the ContEst16S algorithm, which also revealed that the Kf1 genome sequence was contaminated with DNA sequences from *Staphylococcus epidermidis*.

The TypeMat genome-assisted taxonomic classifier at the Microbial Genomes Atlas (MiGA; https://aau.microbial-genomes.org) was used to complement this analysis (accessed on 30 July 2025) (Table S1b) (45). Except for isolate Cluster_DBSCAN, which most likely belongs to a species of the genus *Paracidovorax* not represented in the MiGA database, all classifications with respect to *Xylophilus* and *Xenophilus* were confirmed. The four wastewater metagenome assemblies were classified by TypeMat as intermediate-quality assemblies based on estimates of the redundancy, completeness, and contamination (in contrast to all the other sequences, which were of high or excellent quality) and assigned to the genera *Acidovorax* (Go_Prim_bin_55, Gw_Prim_bin_50) and *Variovorax* (Gw_UH_bin_252). Surprisingly, strain Kf1 was reported as a new species of the genus *Xylophilus*, which might be due to the fact that MiGA only considers contigs with at least 1,000 bp for classification. In the case of strain Kf1, only 116 of 1,939 contigs were considered, and sequence information corresponding to 1,823 contigs (775,918 bp) was omitted, which might explain the classification as a *Staphylococcus* strain by TYGS. Hence, 10 genome sequences were classified as *Xylophilus* by both tools; one was only supported by TYGS (Cluster_DBSCAN), and another was only supported by MiGA (Kf1).

To clarify the sequence relationships among the various genomes, genome-wide ANI was calculated (Table S2a) (47). Four metagenome-assembled genome sequences, originating from municipal wastewater samples, were omitted due to poor quality and apparent incompleteness as evidenced by their small sizes and quality metrics (Table S1b). This analysis suggests that the 12 *Xylophilus* strains, including Cluster_DBSCAN and Kf1, belong to 10 different species. CFBP 1192 and CECT7646, with genome sequences sharing more than 99.99% sequence identity, are both equivalent to the *X. ampelinus* type strain of the species held in two different collections. The genome sequences of isolates SP51_3 and SP210_2 were more than 97.8% identical, suggesting that they both correspond to the same species. The three sequences that appeared to belong to the genus *Xenophilus* (strains/isolates BgEED, CCH5-B3, ERR594292) were more than 98.5% identical to each other, indicating that they belong to the same species. The most closely related of these three strains is *Xenophilus aerolatus*, whose type strain, DSM 19424, shares more than 91.9% genome-wide ANI with each of its genomes.

Digital DNA-DNA hybridization (DDH) was performed to complement the ANI calculations (Table S2b) (79). This analysis confirmed the conclusion that the 12 *Xylophilus* genomes correspond to 10 species. The DDH of the genome sequences of isolates SP51_3 and SP210_2 was estimated at 86%, well above the generally accepted species boundary of 70% (80). Likewise, strains/isolates BgEED, CCH5-B3, and ERR594292 had pairwise DDH values of at least 96% to each other and were most similar to the *X. aerolatus* type strain DSM 19424 with 45%–46% DDH values. Taken together, these analyses established a well-curated data set of 12 genome sequences from 11 different strains for further analyses.

### Phylogenetic tree and presence of flagellar and type III secretion genes

**Fig 1:**
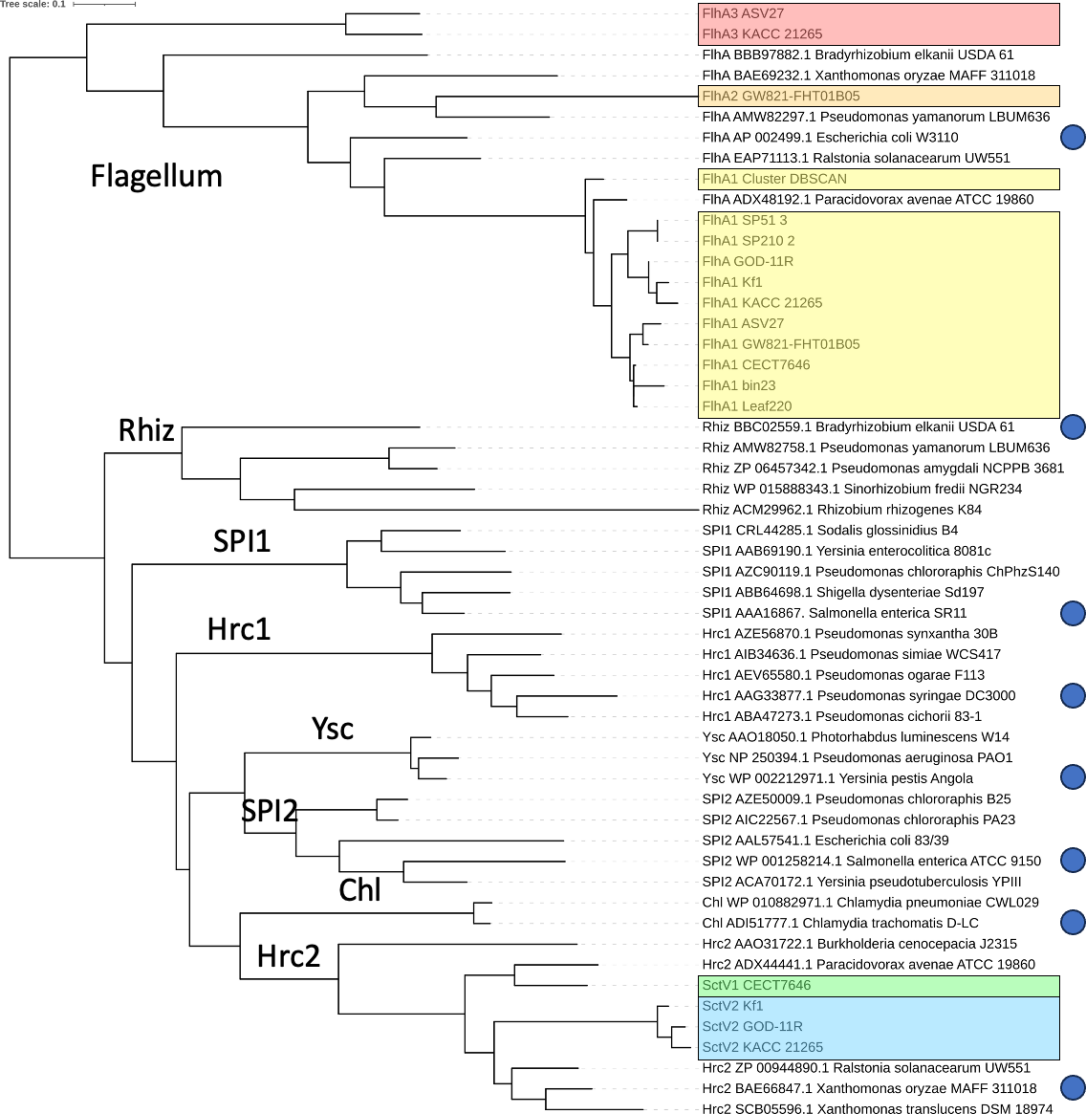
Phylogenetic tree of representative SctV/FlhA proteins and homologs from *Xylophilus. Xylophilus* sequences are boxed with the three flagellar proteins in yellow (type 1), orange (type 2), and red (type 3), and the Sct proteins in green (T3SS#1) and blue (T3SS#2). Strain CFBP 1192 was omitted because it is a clone of the *X. ampelinus* type strain CECT7646. Sequences that were used to identify SctV/FlhA homologs in proteomes of *Xylophilus* are indicated by a blue dot.

Since many Gram-negative plant-associated bacteria rely on a T3SS for pathogenicity or symbiosis, and because most of the *Xylophilus* strains were found to be associated with plants, we hypothesized that these bacteria encode such a secretion system in their genomes. The genome sequences were scrutinized for the presence of T3SS-related sequences based on two of the most conserved proteins, SctV (a homolog of the flagellar FlhA) and SctU (a homolog of the flagellar FlhB) (17, 22, 81). One representative sequence from each of the seven T3SS classes was used as the query, as well as one flagellar homologous sequence, i.e., seven SctU and FlhB sequences, and seven SctV and FlhA sequences (Fig. 1). BLASTP searches against the predicted proteomes of 11 *Xylophilus* strains identified 19 hits for SctU/FlhB and 19 hits for SctV/FlhA. Each strain contained one to three paralogs of each protein. Of the 19 hits, four represent paralogous doublets derived from the two *X. ampelinus* type strain genomes. Since the genome sequence of isolate SP51_3 was not annotated, we used TBLASTN to find homologs of SctU and SctV.

A multiple sequence alignment of the 20 *Xylophilus* SctV homologs revealed that they fall into five groups (Data S1). A phylogenetic tree reconstructed based on the multiple sequence alignment of the 20 sequences, as well as representatives of flagellar and T3SS sequences (17), further supported that these sequences form five clades, which we tentatively called SctV1, SctV2, FlhA1, FlhA2, and FlhA3 (Fig. 1). Members of both SctV clades aligned within the Hrp2/Hrc2 class of SctV proteins. Members of clade FlhA1 and the FlhA2 sequence clustered within the flagellar class. Finally, the two FlhA3 sequences formed a sister clade of the flagellar class, suggesting that they belong to flagellar systems. A similar result was obtained when comparing representative SctU/FlhB sequences with the homologs from *Xylophilus* (Fig. S1).

**Fig 2:**
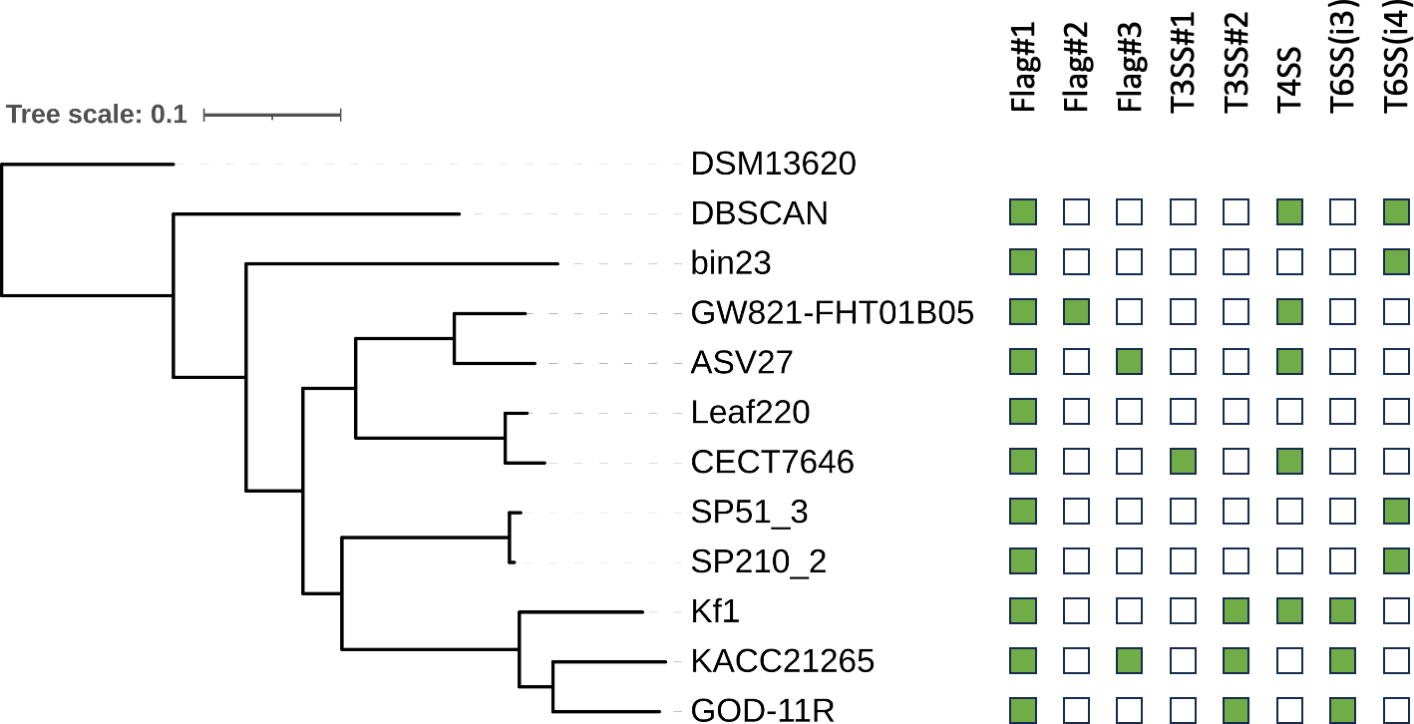
Presence of flagellar and secretion systems in strains of *Xylophilus*. The phylogenetic tree was reconstructed from the core proteome using M1CR0B1AL1Z3R, and is based on 644 proteins with 231,120 aligned amino acids. All nodes are supported with 100% bootstrap values. The genome of the *Xenophilus* type strain DSM 13620 was used to root the tree. Presence of flagellar and secretion systems, as deduced from homology to the conserved *sctV*/*flhA* gene and supported by TXSScan, SeCreT4, and SeCreT6 analyses, is indicated by green squares and absence by open squares.

To examine the distribution of the various flagellar and secretion systems among the *Xylophilus* strains, a core-genome-based phylogenetic tree was computed using the M1CR0B1AL1Z3R suite (Fig. 2) (56). All strains were found to possess the type-1 flagellum comprising FlhA1 and FlhB1. The second flagellar type with FlhA2 and FlhB2 was only found in strain GW821-FHT01B05. The distribution of the third flagellar type with FlhA3 and FlhB3 was patchy and did not follow phylogenetic descent. The Hrp2/Hrc2 T3SS with SctU1 and SctV1 was only found in the *X. ampelinus* strain (CECT7646/CFPB 1192). The second Hrp2/Hrc2 T3SS type was present in a lineage comprising the strains Kf1, GOD-11R, and KACC 21265.

We also evaluated the presence of two additional multi-protein secretion systems, the type IV and type VI secretion systems (T4SS, T6SS). The presence of these systems was predicted using SeCreT4 and SeCreT6 (59, 60). The i3-class T6SS was found in the same clade as the type-2 T3SS, whereas the i4-class T6SS was found in three genetic lineages comprising DBSCAN, bin23, and the two similar isolates SP51_3 and SP210_2. Gene clusters encoding T4SS were found in five strains, DBSCAN, Kf1, GW821-FHT01B05, ASV27, and CECT7646/CFPB 1192, and their presence-absence pattern suggests multiple gain and loss events of this system along the phylogenetic tree (Fig. 2). These results were confirmed using the TXSScan program (Table S3) (58).

Hrp2/Hrc2-class T3SSs are under transcriptional control of HrpX, an AraC-type regulator that binds to a conserved PIP box. When a matching −10 promoter element is in a proper distance of 30 to 32 base pairs, genes are transcribed, most of them encoding components of the T3SS, T3Es, and type II-secreted cell wall-degrading enzymes. Indeed, perfect PIP patterns with the consensus sequence TTCGB-N_15_-TTCGB-N_30-32_-YANNNT were found in the promoters of the Hrp2/Hrc2 T3SS gene clusters, in front of *sctD* and *sctU* in strain CECT7646 and in front of *sctQ* in strain KACC 21265 (Table 2). Nine of the 16 PIP patterns in the genome of strain CECT7646 were found upstream of T3E genes (*hopQ*, *ripC*, *ripE*, *ripAC*, *ripAQ*, *xopD*, *xopZ*, *xopAI*, *xopBG*), and one upstream of a pectate lyase gene (*ripW*). Two genes, DFQ15_RS08770 and DFQ15_RS08785, had only a homolog in *Sphingomonas* sp. NCPPB 2930 in a region with a similar T3SS.

**TABLE 2:** Candidate HrpX-regulated promoters of *X. ampelinus* strain CECT7646 and *X. rhododendri* strain KACC 21265

Locus_tag	Old	PIP box sequence*^a^*	Distance 1 (bp)*^b^*	–10 motif	Distance 2 (bp)*^c^*	Homolog/remark*^d^*
**Strain**		**CECT7646**				
DFQ15_RS16870	N.A.	**ttcgt**cagcctgcacatcgg**ttcgt**	30	**ta**atg**t**	29	XopBG
DFQ15_RS01080	DFQ15_101225	**ttcgc**gctgctggcccagct**ttcgc**	31	**ca**cac**t**	59^§^	HopQ
DFQ15_RS02885	DFQ15_102289	**ttcgc**tccgtcatgatcgac**ttcgt**	31	**ca**cac**t**	95	XopZ
DFQ15_RS05435	DFQ15_10577	**ttcgt**tctcataccgcccgg**ttcgg**	31	**ca**cgg**t**	−525	Antisense
DFQ15_RS17705	DFQ15_105103	**ttcgt**ttgtcatgtcacatc**ttcgc**	30	**ta**tca**t**	236	RipC
DFQ15_RS05805	DFQ15_10628	**ttcgg**ccccgcgcccacggc**ttcgt**	31	**ta**agg**t**	85^§^	RipAQ
DFQ15_RS07770	N.A.	**ttcgc**tttggccgcgaaata**ttcgc**	30	**ca**tcc**t**	33	XopD
DFQ15_RS08655	DFQ15_1117	**ttcgt**ttcgcgcaacatcgg**ttcgc**	30	**ca**tcc**t**	45	RipW
DFQ15_RS08715	DFQ15_11120	**ttcgc**aaatcatgaaccggc**ttcgc**	31	**ta**gtc**t**	74^§^	SctD
DFQ15_RS08730	DFQ15_11123	**ttcgc**accgtgggccaccgc**ttcgg**	30	**ta**gat**t**	66	SctU
DFQ15_RS08770	DFQ15_11131	**ttcgg**tattcatgcaagttc**ttcgt**	30	**ta**gcc**t**	30	Hpa3
DFQ15_RS08785	DFQ15_11134	**ttcgc**aaatcgcggatcgcc**ttcgc**	30	**ta**ttc**t**	66	Hpa2
DFQ15_RS10885	DFQ15_11536	**ttcgt**atagcagggaatatg**ttcgc**	31	**ca**cca**t**	120	RipAC
DFQ15_RS11495	DFQ15_11681	**ttcgg**ctacgcgcatgctgt**ttcgc**	31	**ca**aga**t**	102	XopAI
DFQ15_RS14030	DFQ15_12414	**ttcgc**ccggacgcatggcac**ttcgg**	31	**ca**atc**t**	74^§^	RipE
DFQ15_RS15225	DFQ15_12925	**ttcgc**caggcagtcgggcgg**ttcgg**	31	**ca**ccg**t**	−1,172	Within CDS
						
**Strain**		**KACC 21265**				
GT347_RS00670	GT347_00670	**ttcgg**ctgggtttgacggac**ttcgc**	31	**ta**cgc**t**	28	2 paralogs
GT347_RS00990	GT347_00990	**ttcgt**cgcacggcagcagcc**ttcgt**	31	**ta**gag**t**	27	
GT347_RS02290	GT347_02290	**ttcgt**gagaaacggtggcca**ttcgg**	31	**ca**cag**t**	30	9 paralogs
GT347_RS02345	GT347_02345	**ttcgg**ggcgaacaacagtga**ttcgc**	31	**ca**cag**t**	31	9 paralogs
GT347_RS02675	GT347_02675	**ttcgt**gcccgcgcggtcgtt**ttcgc**	30	**ta**ggc**t**	46	No homologs
GT347_RS03520	GT347_03520	**ttcgc**cttcggtcctgcagc**ttcgg**	31	**ca**agc**t**	265	
GT347_RS04255	GT347_04255	**ttcgc**atggaacgggcggca**ttcgt**	31	**ca**gag**t**	30	5 paralogs
GT347_RS04260	GT347_04260	**ttcgc**aataaacaagcggca**ttcgg**	31	**ca**aag**t**	31	5 paralogs
GT347_RS04265	GT347_04265	**ttcgc**gagggccagggagct**ttcgt**	31	**ca**aag**t**	32	5 paralogs
GT347_RS05075	GT347_05075	**ttcgt**aatgcacgggggata**ttcgc**	30	**ca**aag**t**	30	5 paralogs
GT347_RS09625	GT347_09625	**ttcgc**ttccgctgcgcggtc**ttcgc**	30	**ca**aag**t**	29	No homologs
GT347_RS10730	GT347_10730	**ttcgc**ctcggcacacggata**ttcgc**	32	**ca**aaa**t**	36	
GT347_RS12520	GT347_12520	**ttcgc**gtccggcccgaggca**ttcgg**	31	**ca**gca**t**	49	No homologs
GT347_RS14590	GT347_14590	**ttcgg**gtgattcgatctgca**ttcgg**	31	**ca**tct**t**	29	XopN
GT347_RS15795	GT347_15795	**ttcgc**cgcggcacggccggc**ttcgc**	31	**ca**tcg**t**	33	13 paralogs
GT347_RS15815	GT347_15815	**ttcgc**cgcagcggcagcggc**ttcgc**	31	**ca**tct**t**	38	13 paralogs
GT347_RS15845	GT347_15845	**ttcgc**cacgggggtgccggc**ttcgc**	31	**ca**tcg**t**	30	13 paralogs
GT347_RS15855	GT347_15855	**ttcgc**tcgggcgtggcggtt**ttcgc**	30	**ca**tga**t**	35	2 paralogs
GT347_RS15860	GT347_15860	**ttcgt**ttgcccagcctgttg**ttcgg**	32	**ca**cca**t**	145^§^	2 paralogs
GT347_RS17215	GT347_17215	**ttcgc**cgcggcgtgccgggt**ttcgg**	30	**ca**tat**t**	38	No homologs
GT347_RS17325	GT347_17325	**ttcgg**ccgtgggcccaggca**ttcgt**	31	**ca**gca**t**	33	No homologs
GT347_RS17385	GT347_17385	**ttcgg**tcgggatgaccgtct**ttcgg**	32	**ta**gcg**t**	33^§^	2 paralogs
GT347_RS18105	GT347_18105	**ttcgg**tttcaccgacgggat**ttcgc**	31	**ca**cat**t**	82	No homologs
GT347_RS18300	GT347_18300	**ttcgc**ctgcggccgtgcgtt**ttcgg**	30	**ca**aca**t**	246	8 paralogs
GT347_RS18380	GT347_18380	**ttcgc**gggatgcggagagcg**ttcgt**	31	**ca**gag**t**	31	No homologs
GT347_RS18405	GT347_18405	**ttcgg**cggcggcggcgcggg**ttcgc**	31	**ca**ccg**t**	41	
GT347_RS18445	GT347_18445	**ttcgt**ggcccggggcgcgga**ttcgc**	32	**ta**cct**t**	7 (73^§^)	RipL
GT347_RS19325	GT347_19325	**ttcgc**gcgcggccgcagcgc**ttcgc**	30	**ca**aga**t**	25	No homologs
GT347_RS19630	GT347_19630	**ttcgc**attcgccgccacaac**ttcgc**	31	**ga**tca**t**	40	No homologs
GT347_RS19635	GT347_19635	**ttcgc**ctggcatcgcaaggt**ttcgg**	31	**ta**cgc**t**	32	No homologs
GT347_RS19640	GT347_19640	**ttcgc**atgcggtgacgacgc**ttcgc**	30	**ta**cgc**t**	47	SctQ
GT347_RS23280	GT347_23280	**ttcgg**tcgcggccccggtgc**ttcgg**	31	**ca**cag**t**	42	No homologs
GT347_RS23660	GT347_23660	**ttcgc**caccgcgcacggtgc**ttcgg**	31	**ca**ggc**t**	31	2 paralogs
GT347_RS24100	GT347_24100	**ttcgg**acgaaggccgacgac**ttcgc**	32	**ta**ggc**t**	175	No homologs
GT347_RS26130	GT347_26130	**ttcgg**cgcggctgcgggcca**ttcgt**	31	**ca**cgc**t**	7 (46^§^)	No homologs

^a^ PIP box half-sites and conserved nucleotides of the –10 promoter motif are shown in bold.

^b^ Distance in base pairs between the end of the PIP box and the –10 promoter motif.

^c^ Distance in base pairs between the end of the –10 promoter motif and the predicted translational start codon. Cases where the structural annotation was revised, resulting in a new start codon, are indicated by a § sign.

^d^ Homologs of effectors (Xop, Rip), as well as components or helpers of the T3SS (Sct, Hpa), are indicated. For 28 CDS downstream of the PIP box, no homologs were found, or paralogs were only found in the same strain. One gene is encoded in antisense orientation to the PIP box. In another gene, the PIP box is found within the CDS. These two cases are likely false positives and may not belong to the HrpX regulon.

The predicted PIP-box harboring gene repertoire in strain KACC 21265 was different from that of strain CECT7646. Among its 32 genes encoding hypothetical proteins were two candidate T3E genes. Protein QHI99106.1 (locus tag GT347_14590) was 22.90% identical to XopN (WP_011348010.1) over 69% of its sequence, and protein QHI99786.1 (locus tag GT347_18445) was 26.04% identical to RipL (WP_014630780.1) over 80% of its sequence. Notably, 15 genes were found to encode members of strain-specific protein families with unknown function, with up to 13 paralogs (Table S4 and Data S2). All of them are annotated as hypothetical proteins. We used InterProScan to shed light on their potential functions. However, no known protein domain was found for any of them. A signal sequence for secretion was predicted for two members of the 13 homologs of protein family 4. For 13 additional hypothetical proteins beyond the eight protein families, we did not find any homologs using BLASTP searches at NCBI GenBank (Table 2).

### Comparative genomic organization of flagellar and type III secretion gene clusters within *Xylophilus* strains

To better classify the flagellar systems and the T3SSs, we compared their genomic organization. To this end, we manually curated the structural and functional annotation of the genomic regions where the *sctV* and *sctU* genes were found, using BLAST and InterProScan searches.

Strain GW821-FHT01B05 appears to encode two types of flagella in large gene clusters. The gene cluster with *flhA1*, which was found in all *Xylophilus* strains, spans 55 genes in strain GW821-FHT01B05, among them two flagellin genes, three *flh* genes (ABF), 13 *flg* genes (ABCDEFGHIJKLM), 18 *fli* genes (ADEFGHIJKLMNOPQRST), two *mot* genes (AB), and seven chemotaxis genes (ABDRWYZ) (Fig. S2). The second gene cluster with *flhA2* contains 38 genes with only one flagellin gene, two *flh* genes (AB), 15 *flg* genes (ABCDEFGHIJ_2_KLMN), 18 *fli* genes (ADEFGHIJK_2_LMNPQRST), and two *mot* genes (AB). This strain contains six additional chemotaxis genes (ABDRWY), which are, however, encoded in another genomic location, and it remains to be determined whether they correspond to the FlhA2 flagellar system.

Another candidate flagellar system, which includes *flhA3*, was found in strains ASV27 and KACC 21265. This system lacks several of the *flg*, *flh*, and *fli* genes. For this system, no flagellin could be identified. Candidate genes for 9 Flg proteins (ABCDEFGHI), 2 Flh proteins (AB), and 12 Fli proteins (AEFHIJMNOPQR) were predicted. Some of the missing genes, such as *fliG*, *fliK*, and *fliL*, might correspond to the hypothetical proteins that are encoded in the gene cluster (Fig. S2).

**Fig 3:**
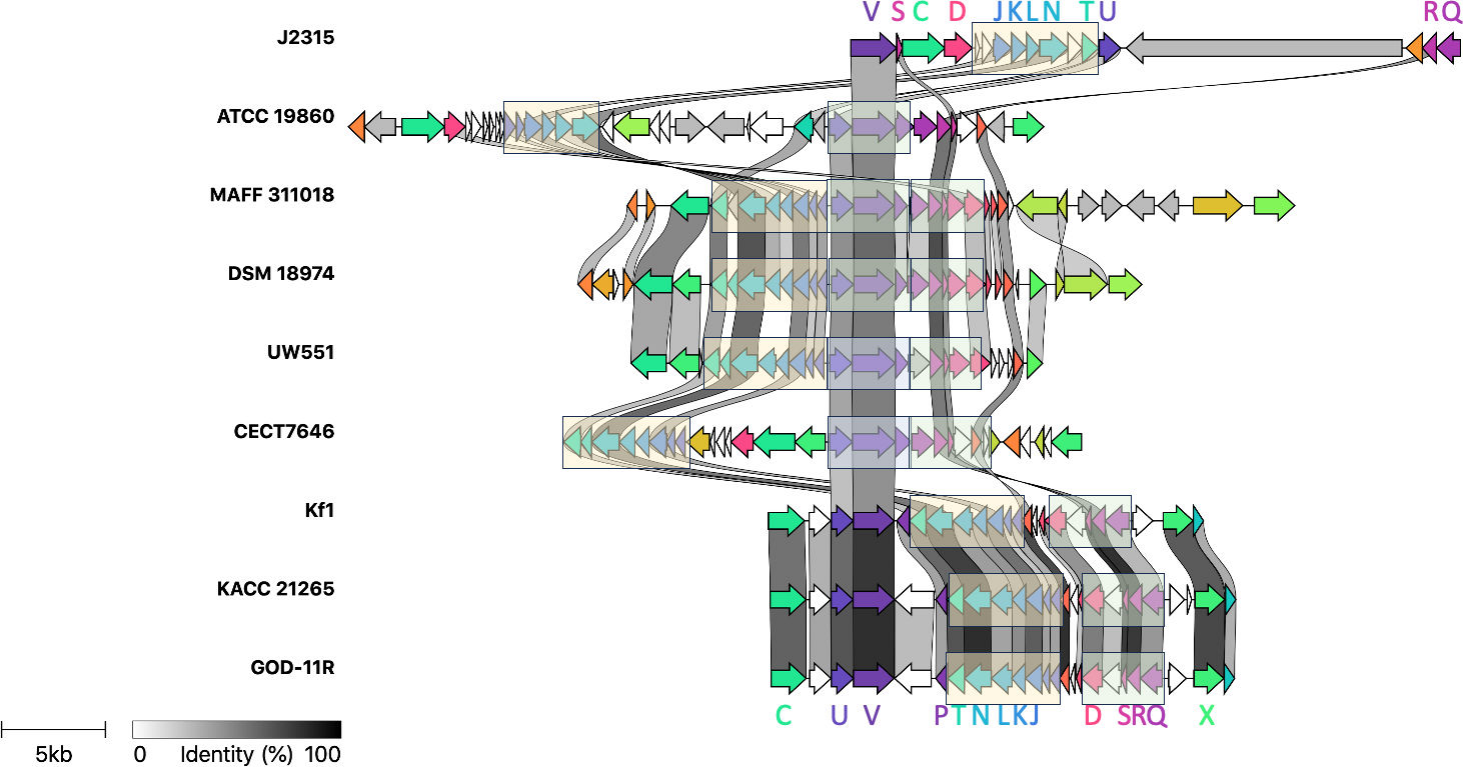
Comparison of *Xylophilus* type III secretion gene clusters. Corresponding regions, extracted from GenBank files, were compared and visualized with Clinker. Two classes of type III secretion gene clusters were observed for *Xylophilus*, as supported by synteny and sequence similarities. Conserved *sct* genes are labeled in the same color as the genes. Blocks with near-perfect synteny are boxed. The following genomic regions are depicted, from top to bottom: QU43_RS64645 (*sctV*) to QU43_RS64560 (*sctQ*) (*Burkholderia cenocepacia* J2315; NC_011001.1), Acav_0545 (*hpa2*) to ACAV_0510 (*hrpX*) (*Acidovorax avenae* ATCC 19860; CP002521.1), XOO0080 (*hpa2*) to XOO0110 (*xopAE*) (*Xanthomonas oryzae* MAFF 311018; AP008229.1), BFN94_RS13565 (*hpa2*) to BFN94_RS21845 (*xopM*) (*Xanthomonas translucens* DSM 18974; NZ_LT604072.1), RRSL_PS12385 (*sctC*) to RRSL_RS12490 (*hpaB*) (*Ralstonia solanacearum* UW551; NZ_AAKL01000026.1), DFQ15_RS08660 (*sctT*) to DFQ15_RS08795 (*hrpX2*) (*Xylophilus ampelinus* CECT7646; NZ_QJTC01000011.1), GN316_15560 (*sctC*) to GN316_15670 (*hrpB7*) (*Xylophilus* sp. Kf1; WPBX01000006.1), GT347_RS19740 (*sctC*) to GT347_RS19620 (*hrpB7*) (*Xylophilus rhododendri* KACC 21265; NP_CP047650.1), R9 X 41_RS04700 (*sctC*) to R9 X 41_RS04585 (*hrpB7*) (*Xylophilus* sp. GOD-11R; NZ_CP137854.1).

Bona fide T3SSs were found in five strains (Fig. 3). For comparison, we included the T3SSs of the Hrp2/Hrc2-representative strains that were used for classification of the SctV/FlhA and SctU/FlhB proteins (Fig. 1 and Fig. S1). The two *X. ampelinus* genomes were found to encode a T3SS with 13 *sct* genes (CDJKLNPQRSTUV), which have a genomic organization that is similar to the one found in clade 1-xanthomonads (*Xanthomonas hyacinthi*, *Xanthomonas theicola*, *Xanthomonas translucens*), in the *Ralstonia solanacearum* species complex, but also in *Collimonas fungivorans*, *Paraburkholderia andropogonis*, and *Uliginosibacterium gangwonense* (82). Notably, the *Xanthomonas hrpB* (eight genes, including *sctJ*, *sctK*, *sctL*, *sctN*, and *sctT*), *hrpC* (three genes, *sctU*, *sctV*, and *sctP*), and *hrpD* (four genes, including *sctQ*, *sctR*, and *sctS*) operons are present (83). As in all these bacteria, the AraC-type transcriptional activator HrpX is encoded within the T3SS gene cluster and likely forms an operon with *sctC*. A second HrpX paralog with 53% sequence identity is encoded at the border of the T3SS gene cluster. The gene upstream of *hrpB1* appears to encode a protein that is distantly related to the putative translocon HrpF/NolX, as supported by PSI-BLAST analysis.

The *Xylophilus* strains KACC 21265, Kf1, and GOD11-R were found to encode another T3SS, including 13 conserved *sct* genes. Their genetic organization is different from that of systems found in other plant pathogens, such as *Acidovorax*, *Pseudomonas*, *Ralstonia*, and *Xanthomonas*. For instance, *hrpB7*, which is located between *sctN* and *sctT* in *Ralstonia* and *Xanthomonas* and upstream of *sctT* in *Acidovorax*, is encoded downstream of *hrpX*. Also, *sctP*, which is usually encoded downstream of *sctV*, is here encoded downstream of *sctT*. No homolog of the putative translocons HrpF/NolX or HpaT was identified. And none of the *Xylophilus* T3SS gene clusters encoded an ortholog of the HrpG response regulator. In addition, we found no evidence for the presence of an HrpG ortholog elsewhere in the genome.

### Prediction of candidate type III effectors in *Xylophilus*

Candidate effectors were predicted using Effectidor II, a pan-genomic AI-based web server (61). Prediction was targeted for the five genome sequences in which T3SSs were found, i.e., strains CECT7646, CFBP 1192, GOD11R, KACC 21265, and Kf1. Effectidor II optionally utilizes information from related strains without T3SSs (ORFs shared between strains with and without T3SSs are less likely to encode effectors). The proteomes of strains ASV27, bin23, GW821-FHT01B05, Leaf220, and SP210_2 were used for this purpose.

For *X. ampelinus* with the SctV1-type T3SS, Effectidor II predicted 36 orthology groups with scores higher than 0.25 (Table 3). Two groups, OG_3401 (NBM50_08240) and OG_1546 (DFQ15_10953), are bona fide orthologs that were separated due to differences in the assembly and structural annotation of the genome sequences. The NBM50_08240 protein variant contains a 52-aa tandem repeat, which is only present once in the DFQ15_10953 variant. The NBM50_08240 protein variant has an N-terminal extension of 378 amino acids compared to the DFQ15_10953 variant. Three orthology groups, OG_3358 (NBM50_04355), OG_3350 (NBM50_03010), and OG_3346 (NBM50_02550), were only structurally annotated in the genome sequence of strain CFBP 1192. Among the 35 bona fide orthology groups, 30 had homologs in a T3E database of plant-pathogenic bacteria, assembled from three individual databases for *Pseudomonas* (Hop’s), *Ralstonia* (Rip’s), and *Xanthomonas* (Xop’s) (84–86) (Table 3). Altogether, the 31 effectors fall into 28 classes, with AvrB, RipTPS, and XopD being represented twice.

**TABLE 3:** Prediction of type III-secreted proteins for SctV1-type T3SS of *X. ampelinus* using Effectidor II^*a*^

OG	Score	Annotation	CECT7646	CFBP 1192	PIP pattern	Effector class	Cov.	Ident.
OG_1112	0.899	Hypothetical protein	DFQ15_105103	NBM50_05555	Yes	RipC	100	67
OG_671	0.898	Hypothetical protein, type III secretion system effector protein	DFQ15_10390	NBM50_03725		XopN	83	60
OG_2572	0.886	Hypothetical protein	DFQ15_12045	NBM50_13085		HopD	75	26
OG_222	0.832	Inosine-uridine preferring nucleoside hydrolase, nucleoside hydrolase	DFQ15_101225	NBM50_02130	Yes	HopQ	100	66
OG_1577	0.819	Hypothetical protein, inositol hexakisphosphate	DFQ15_10984	NBM50_08090		XopH	97	64
OG_1600	0.817	Hypothetical protein	DFQ15_109107	NBM50_07980		XopX	83	63
OG_576	0.801	Hypothetical protein	DFQ15_102289	NBM50_01715	Yes	XopZ	90	41
OG_297	0.800	Avirulence protein, hypothetical protein	DFQ15_1023	NBM50_00260		AvrB	96	64
OG_1753	0.794	Hypothetical protein	DFQ15_11138	NBM50_09320		RipA	56	25
OG_1576	0.787	Hypothetical protein, secretion protein EspV	DFQ15_10983	NBM50_08095		AvrA	94	62
OG_2604	0.735	Hypothetical protein, type III effector HopG1	DFQ15_12111	NBM50_13615		XopAG	90	57
OG_944	0.731	Hypothetical protein	DFQ15_104122	NBM50_04820		XopV	82	62
OG_1116	0.720	Hypothetical protein	DFQ15_105107	NBM50_05530		/^*b*^	/	/
OG_1115	0.703	Hypothetical protein	DFQ15_105106	NBM50_05540		/	/	/
OG_1575	0.695	Avirulence protein AvrBs1, type III secretion AvrA family effector	DFQ15_10982	NBM50_08100		AvrBs1	89	59
OG_2287	0.687	ADP-ribosyltransferase domain-containing protein, NAD:arginine ADP-ribosyltransferase	DFQ15_11681	NBM50_12035	Yes	XopAI	100	53
OG_1573	0.684	Fic family protein, leucine-rich repeat (LRR) protein	DFQ15_10980	NBM50_08110		XopAC	74	32
OG_3401	0.678	C48 family peptidase		NBM50_08240		XopD	97	47
OG_864	0.673	Cation transport regulator ChaC, gamma-glutamylcyclotransferase	DFQ15_10441	NBM50_05235		RipAY	45	43
OG_3308	0.671	Hypothetical protein	DFQ15_1541	NBM50_17140		/	/	/
OG_1103	0.671	Avirulence protein, hypothetical protein	DFQ15_10594	NBM50_05600		AvrB	75	49
OG_92	0.638	Lytic murein transglycosylase	DFQ15_10194	NBM50_02800		HopAJ	96	47
OG_1546	0.549	Type III effector protein XopD	DFQ15_10953			XopD	84	47
OG_906	0.453	Trehalose 6-phosphate synthase, trehalose-6-phosphate synthase	DFQ15_10483	NBM50_05020 (pseudogene)		RipTPS	91	57
OG_1203	0.332	Hypothetical protein	DFQ15_10675	NBM50_06370		XopL	92	33
OG_2795	0.327	Hypothetical protein	DFQ15_12414	NBM50_14825	Yes	RipE	44	29
OG_3358	0.321	Hypothetical protein		NBM50_04355		XopO	87	45
OG_3350	0.317	Hypothetical protein		NBM50_03010		XopBG	79	42
OG_2335	0.293	YopT-type cysteine protease domain-containing protein, YopT-type cysteine protease-like protein	DFQ15_11735	NBM50_12290		HopAR	83	31
OG_1323	0.292	Glycoside hydrolase family 28 protein, polygalacturonase	DFQ15_10769	NBM50_06990		/	/	/
OG_1938	0.289	E3 ligase-like protein (putative virulence factor), hypothetical protein	DFQ15_112108	NBM50_09720		RipV	64	38
OG_1158	0.281	Hypothetical protein	DFQ15_10628	NBM50_06585	Yes	RipAQ	65	33
OG_1549	0.273	Alpha, alpha-trehalose-phosphate synthase (UDP-forming)	DFQ15_10956	NBM50_08225		RipTPS	99	48
OG_2162	0.257	Hypothetical protein, LRR protein	DFQ15_11536	NBM50_11465	Yes	RipAC	27	32
OG_1722	0.256	Pectate lyase, pectate lyase-like protein	DFQ15_1117	NBM50_09160	Yes	RipW	61	37
OG_3346	0.254	Hypothetical protein		NBM50_02550		/	/	/

^*a*^ Best hits with at least 25% coverage are shown. Candidate effectors with a corresponding PIP pattern (Table 2) are indicated. Corresponding effector classes, as revealed by BLASTP searches against a plant-pathogen T3E database, are listed along with the coverage and sequence identity.

^*b*^ /, not applicable.

The situation was much different for the SctV2-type T3SS, which was found in strains GOD-11R, KACC 21265, and Kf1. Effectidor II predicted only six orthology groups with scores higher than 0.25 (Table 4). Four orthology groups had homologs in the T3E database (HopAJ, RipA, RipL, RipTPS), and each strain had two or three of these T3E candidates.

**TABLE 4:** Prediction of type III-secreted proteins for SctV2-type T3SS of *Xylophilus* using Effectidor II^*a*^

OG	Score	Annotation	GOD-11R	KACC 21265	Kf1	Effector class	Cov.	Ident.
OG_92	0.638	Lytic murein transglycosylase	R9 X 41_13525	GT347_03245	GN316_20430	HopAJ	98	49
OG_9901	0.285	Hypothetical protein			GN316_16015	RipA	83	30
OG_1549	0.273	Alpha, alpha-trehalose-phosphate synthase (UDP-forming)	R9 X 41_19915	GT347_17110	GN316_10830	RipTPS	99	48
OG_5313	0.259	Hypothetical protein	R9 X 41_16315			/^*b*^	/	/
OG_7634	0.255	Hypothetical protein		GT347_22755		/	/	/
OG_7234	0.251	Hypothetical protein		GT347_17980		RipL	67	32

^*a*^ Best hits with at least 25% coverage are shown. Corresponding effector classes, as revealed by BLASTP searches against a plant-pathogen T3E database, are listed along with the coverage and sequence identity.

^*b*^ /, not applicable.

### Confirmation and structural modeling of predicted type III effectors

We next aimed to experimentally verify the secretion signal of novel effector candidates. Based on the high Effectidor II scores, BLASTP and InterProScan analyses, we focused on three orthology groups, OG_1116, OG_1115, and OG_3308, with Effectidor II scores of 0.720, 0.703, and 0.671, respectively (Table 3). Members of the other orthology groups had a substantially lower Effectidor II score (below 0.3) and had either no homologs beyond *Xylophilus* (OG_5313, OG_7634) or well-defined homologs that are not considered to be T3Es (OG_1323, OG_3346) (Tables 3 and 4). Members of OG_1323 are annotated as a glycoside hydrolase family 28 protein or a polygalacturonase, proteins that are typically secreted by the type II secretion pathway. Indeed, SignalP predicts a Sec/SPI-type Signal Peptide (Sec/SPI) with a likelihood of 0.999 for the 629-aa protein PYE78419 with the locus ID DFQ15_10769. OG_3346 has close homologs with more than 60% sequence identity that are annotated as cytosine-specific methyltransferases and that are encoded on *Acidovorax* bacteriophages (UYL85549.1, UYL85448.1). This observation prompted us to consider that the locus NBM50_02550 may belong to a prophage. To test this, we used geNomad to assess whether this locus is part of a mobile genetic element (67), which was indeed the case. NBM50_02550 corresponds to one out of 58 ORFs that are encoded on a predicted 46-kb prophage (region 142964 to 188749 of contig QJTC01000001.1). For these reasons, we did not further study the orthology groups OG_5313, OG_7634, OG_1323, and OG_3346.

Three orthology groups, OG_1116 (DFQ15_105107, NBM50_05530), OG_1115 (DFQ15_105106, NBM50_05540), and OG_3308 (DFQ15_1541, NBM50_17140), corresponded to proteins that are similar to each other. However, a multiple sequence alignment revealed that the proteins belonging to OG_3308 lack the C terminus, which can be explained by the fact that the gene is located at the end of the contigs in both strains, CFBP 1192 (contig ID: JAMOFZ010000052.1) and CECT7646 (contig ID: QJTC01000054.1). The full-length versions of OG_1116 and OG_1115 have amino acid sequences that are 58% identical to each other. The C-terminally truncated version of OG_3308 is 54% identical to OG_1116 and 80% identical to OG_1115.

In total, there are 12 homologous protein variants deposited at NCBI, corresponding to the two strains, CECT7646 and CFBP 1192, each with three paralogs, and each paralog is either annotated by the submitters and re-annotated by NCBI (RefSeq versions predicted with GeneMarkS-2+). Their multiple sequence alignment revealed that three different translation start codons were used (Data S3). Based on sequence comparisons and the presence of a putative Shine-Dalgarno sequence, GGAG, with a distance of six base pairs to the ATG translation initiation codon, we considered the protein versions starting with MKDLYPF (Met-Lys-Asp-Leu-Tyr-Pro-Phe) as the most likely ones. The G+C content of the coding sequences is 56.7% for OG_1115, 56.9% for OG_1116, and 53.8% for OG_3308, compared to an overall G+C content of the genome of 67.8%. Such atypical G+C content can be taken as evidence for horizontal gene transfer, a process that is known to be involved in shaping the T3E repertoires of bacterial pathogens (87).

**Fig 4:**
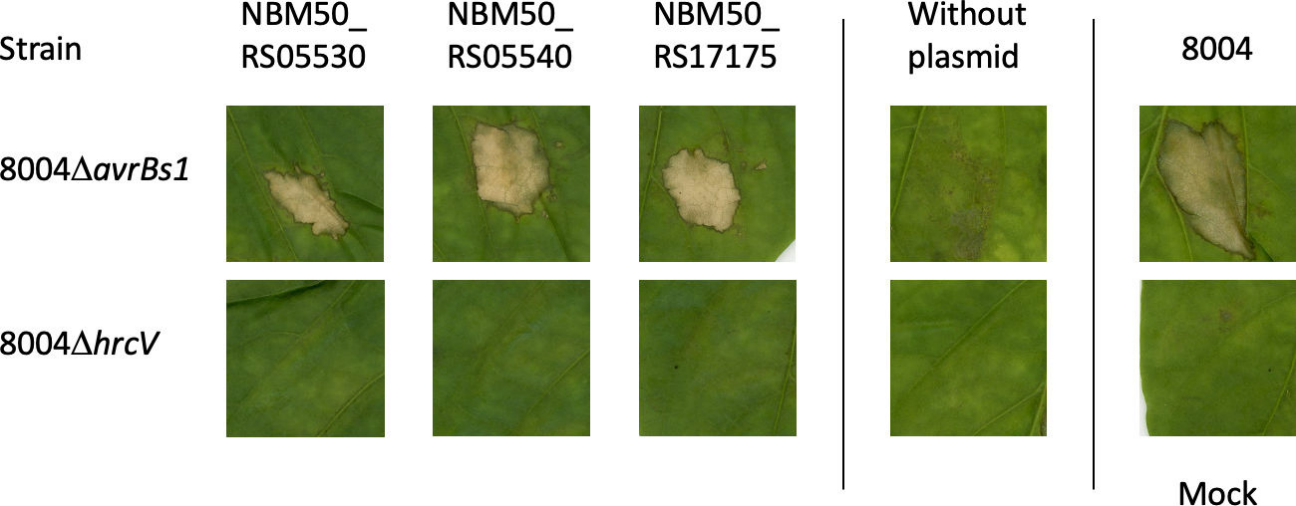
Translocation assays of novel T3E AvrBs1 reporter protein fusions. Bacterial *Xanthomonas* cells were inoculated into the leaves of pepper cv. ECW-10R using a needleless 1-mL plastic syringe. Strain 8004, which carries a copy of the *avrBs1* gene and can elicit HR on non-host pepper cv. ECW-10R (38) was the positive control. Strains 8004Δ*avrBs1*, 8004Δ*hrcV,* and mock inoculation with the buffer served as negative controls. The results presented are from a representative experiment, with similar results obtained in all other experiments.

We employed a reporter fusion system where the candidate effector’s type III secretion signal is translationally fused to the defense-triggering domain of AvrBs1, a T3E from *Xanthomonas campestris* (39). Our chimeric constructs consisted of the 100 base pairs upstream of the most likely translation initiation codon and the first 100 codons of the CDS, fused to the *avrBs1* CDS covering codons 59 to 445. When introduced into the *X. campestris* reporter strain 8004Δ*avrBs1*, bacteria expressing AvrBs1 reporter fusions triggered a *Bs1*-dependent hypersensitive response in pepper plants of the cultivar ECW-10R (Fig. 4). In contrast, plants showed no reaction upon infiltration with bacteria that expressed the chimeric proteins but were mutated in the essential T3SS gene *hrcV*, thus demonstrating that the N-terminal 100 amino acids functioned as a type III secretion/translocation signal.

To gain insight into the function and conservation of the novel T3Es, we used BLASTP to identify homologous sequences in the ClusteredNR database at NCBI GenBank. Sequences corresponding to OG_1115 and OG_1116 had 70 unique hits with an E-value lower than 10^−10^ (Table S5). Most of these sequences were annotated as hypothetical proteins, and they originated from *Pseudomonas savastanoi*, *Pseudomonas amygdali*, *Acidovorax* sp., *Pseudomonas syringae*, *Xanthomonas fragariae*, *Xanthomonas hydrangeae*, *Paraburkholderia aspalathi*, *Ralstonia* sp., and *Bradyrhizobium* sp., thus including both pathogenic and symbiotic bacteria. Notably, a few of the hits were annotated as C48 family peptidase or Ulp1 family isopeptidase. We then applied CD-Hit with a sequence identity threshold of 0.5 and a word size of 2 to cluster these sequences and established a set of 21 representative sequences. For functional predictions, these sequences were scanned at InterProScan (Table S6). Five of the sequences had hits with InterPro entries corresponding to the Ulp1 protease family. However, the portions of OG_1115 and OG_1116 that aligned with these sequences did not correspond to the Ulp1 protease motifs.

To obtain further insight into the molecular function of the novel T3Es, we modeled the 3D structure of the two full-length versions with AlphaFold3 (Fig. S3) (63). The predicted template modeling scores were 0.39 for OG_1115 and 0.36 for OG_1116. Notably, the N-termini were largely predicted to be disordered (135 aa for OG_1115 and 109 aa for OG_1116), as was the case for the InterProScan predictions (regions 24–67 and 88–124 for OG_1115, region 16–116 for OG_1116), or they would include alpha-helices with very low confidence (pIDDT scores < 50). Structural predictions focusing on the apparently folded C-terminal domains, OG_1115_149-636_ and OG_1116_117-598_, improved the overall confidence of the predictions slightly to 0.42 and 0.40, respectively. This is consistent with previous studies showing that many T3Es possess intrinsically disordered N-terminal regions. For example, Gazi et al. (88) reported that 75% of *Pseudomonas syringae* pv. *tomato* T3Es exhibit a significant propensity for structural disorder within their first 50 N-terminal residues. In addition, LeBlanc et al. (89) showed that efficient translocation requires T3Es to be mechanically labile. Their findings support a model in which unfolding and secretion initiate at the N-terminus, with the intrinsic disorder of effector N-terminal regions likely facilitating this process by reducing the energetic barrier to unfolding initiation.

The predicted models were then used to find structural homologs, using FoldSeek (64). This way, a few T3Es (e.g., HopAV1 from *Pseudomonas amygdali*, HsvG, and HsvB from *Pantoea agglomerans*), but also several ubiquitin-like protease domain-containing proteins from rhizobia (*Mesorhizobium*, *Bradyrhizobium*, *Rhizobium*, and *Sinorhizobium*) were found (Table S7). Most hits in other plant pathogens beyond *Xylophilus* had lower E-values (between 2.3 × 10^−59^ and 1.7 × 10^−11^) than hits in symbiotic bacteria (E-values above 3.6 × 10^−12^). Thus, additional experimental work is needed to better elucidate the function(s) of these newly identified effectors.

## DISCUSSION

Based on genomic data, we aimed to perform a genus-wide prediction of type III secretion and flagellar systems in *Xylophilus*, including the grapevine pathogen *X. ampelinus*. First, we curated the data available in NCBI to resolve taxonomic uncertainties. We found 19 genome sequences at NCBI GenBank, 6 of which were annotated as *X. ampelinus* and 1 of which was annotated as *X. rhododendri*. Based on average nucleotide identities, however, only two strains (CECT7646, CFBP 1192) corresponding to the same original isolate belong to the species *X. ampelinus*, while the genomes of BgEED09, bin23, CCH5-B3, and ERR594292 do not belong to this species. Based on ANI and DDH, isolates BgEED09, CCH5-B3, and ERR594292 do not even belong to the genus *Xylophilus*, since they are genetically closer to three type strains of the genus *Xenophilus* (76–78). Only the isolate bin23 appears to belong to the genus *Xylophilus* and may represent a new, yet uncharacterized species within this genus. Altogether, the remaining 16 genome sequences would correspond to 10 different species, among which only 2, *X. ampelinus* and *X. rhododendri*, have been formally described (3, 4).

Since many Gram-negative pathogenic and symbiotic bacteria rely on a T3SS and its set of secreted effectors for their interaction with eukaryotic organisms, we scrutinized all 12 good-quality genome sequences of the genus *Xylophilus* for the presence of such a protein secretion system and the evolutionarily related flagellar system. Firstly, we found three different flagellar systems in the genus, one of which was present in all 12 strains and is thus vertically inherited. The other two flagellar systems were only detected in one or two strains, and it remains to be analyzed whether the systems were lost in the other strains of the genus or if they were acquired by horizontal gene transfer in this subset of strains.

Based on sequence similarities and supported by synteny, we identified two types of T3SSs, both of which are classified as Hrp2/Hrc2-type T3SSs. Genes of this secretion system are under transcriptional control of the AraC-type transcriptional activator HrpX in *Acidovorax* and *Xanthomonas* and its ortholog HrpB in *Burkholderia* and *Ralstonia* (25, 90). We identified homologs of HrpX/HrpB in the T3SS gene clusters of *Xylophilus*, and we also identified the HrpX/HrpB-binding motif, the *hrpII*/PIP box (26, 27), in the promoters of several T3SS operons in *Xylophilus*, suggesting a similar regulatory mechanism to that in the well-studied plant pathogens mentioned above. However, we were unable to identify a bona fide ortholog of the upstream regulatory component HrpG (36), although each of the various *Xylophilus* genomes encodes dozens of response regulators. Notably, we found two paralogs of HrpB/HrpX genetically linked to the T3SS gene cluster in *X. ampelinus*, one of which is encoded immediately upstream of *sctC* and the other, which has 53% sequence identity, is encoded seven genes downstream of *hpaB*. It remains to be determined how transcription of the *hrpB*/*hrpX* genes is controlled in *Xylophilus*.

T3Es, which are secreted by the T3SS, need assistance in entering the eukaryotic target cell. For Hrp2/Hrc2-type T3SS, two types of putative translocons have been described, HrpF/NolX and HpaT (82, 91). The gene upstream of the *X. ampelinus hrpB1* gene appears to encode a protein that is distantly related to the putative translocon HrpF/NolX, as supported by PSI-BLAST analysis and structural modeling (data not shown). However, we were unable to identify a putative translocon protein for the other T3SS, which is present in GOD11-R, Kf1, and KACC 21265, and it remains to be determined whether this T3SS injects effector proteins into eukaryotic target cells.

The presence of HrpB/HrpX homologs prompted us to predict, using a very conservative approach, genes that are very likely under transcriptional control of HrpB/HrpX and thus co-regulated with the T3SS genes. The power of this approach is underlined by the fact that 4 out of 16 predicted promoters in *X. ampelinus* control the expression of components or helpers of the T3SS (Hpa2, Hpa3, SctD, SctU) and another 10 promoters control candidate T3E genes (*hopQ*, *ripC*, *ripE*, *ripW*, *ripAC*, *ripAQ*, *xopD*, *xopZ*, *xopAI*, *xopBG*). Two additional patterns were found within a CDS or in the antisense direction of a gene and likely represent false positives. For *X. rhododendri*, 35 HrpB/HrpX-regulated promoters were predicted, among which three are found in a region of 1,040 bp upstream of the *sctQ* CDS, and two belong to genes with remote sequence homology to T3Es (XopN, RipL). Surprisingly, the remaining 30 promoters would all control the expression of hypothetical proteins, most of them without homologs beyond *X. rhododendri* and many of them belonging to gene families with several, sometimes distantly related, paralogs. It remains unclear whether these proteins represent T3Es or play roles in interactions with other organisms.

Using a pan-genomic AI-based algorithm, we predicted candidate T3Es for *X. ampelinus* and *X. rhododendri*. In this way, we identified 31 homologs of Hop, Rip, and Xop proteins from *Pseudomonas*, *Ralstonia,* and *Xanthomonas* in *X. ampelinus*, which collectively form a large repertoire of candidate T3Es. This situation resembles that of other highly virulent plant-pathogenic bacteria, which deploy their effector repertoires, also termed effectome or effectorome, to successfully colonize and manipulate their host plants (92, 93). By contrast, we only predicted four Hop or Rip homologs in *X. rhododendri*, which raises questions about its pathogenic potential. Indeed, strain KACC 21265 was isolated from a rhododendron flower, and there is no evidence that it would cause symptoms on rhododendron or other plants.

The taxonomic history of *X. ampelinus* reflects the broader evolution of bacterial systematics—from early reliance on phenotype and host association to modern genetic and phylogenetic methods. The pathogen causing bacterial necrosis of grapevine was formally described in 1969 by Panagopoulos as *Xanthomonas ampelina*, which was assigned to *Xanthomonas* largely because it looked and behaved like other xanthomonads, such as plant pathogenicity and slow growth at ambient temperatures, not because of evolutionary evidence. The bacterium was classified in the genus *Xanthomonas* as it is an aerobic, non-sporing, Gram-negative rod with one polar flagellum, produces a yellow water-insoluble pigment, and metabolizes carbohydrates oxidatively (94). Yet, the grapevine pathogen showed many significant physiological and biochemical differences in comparison with other described species of *Xanthomonas*. Using rRNA–DNA hybridization and enzymatic characteristics analyzed by the Analytical Profile Index system, which was commercialized by bioMérieux, Willems et al. (3) later demonstrated that *X. ampelina* was not phylogenetically related to *Xanthomonas*. Its closest relatives were in Betaproteobacteria, not the Gammaproteobacteria where *Xanthomonas* belongs. Against this background, it is interesting to note that the effector repertoire of *Xylophilus ampelinus* bears a strong resemblance to the repertoires of other plant-pathogenic bacteria, including *Xanthomonas*.

Three of the predicted T3Es of *X. ampelinus*, for which no homolog was found in T3E databases of *Pseudomonas*, *Ralstonia*, and *Xanthomonas*, were found to possess a functional type III secretion signal that enabled the secretion and translocation of an AvrBs1 reporter protein from *Xanthomonas* into pepper plants. Rather than using *X. ampelinus* itself, we used *X. campestris* as a surrogate system to test the secretion signals of *Xylophilus* candidate effectors. This choice was due to the absence of suitable reporter plasmids, hosts with corresponding resistance genes, mutants in *Xylophilus*, as well as the lack of established genetic transformation protocols. Interestingly, the surrogate system proved effective in studying effector candidates from other taxa. Our work shows that heterologous expression in a gamma proteobacterium can be used to validate candidate effectors from a beta proteobacterium.

There are a few examples of such trans-class secretion experiments. For instance, *Xanthomonas euvesicatoria* pv. *euvesicatoria* (syn. *X. campestris* pv. *vesicatoria*) was able to secrete PopA, a type III-secreted protein from *R. solanacearum* (95). While these secretion experiments were tested for secretion using the same class of T3SS, here the Hrp2/Hrc2 class, other studies have shown that T3Es can even be secreted by a different T3SS class, at least when overexpressed. For instance, *X. euvesicatoria* pv. *euvesicatoria* could secrete AvrB, a Hrc1-class type III-secreted avirulence protein from *P. syringae* pv. *glycinea*, and YopE, a Ysc-class type III-secreted cytotoxin of the mammalian pathogen *Yersinia pseudotuberculosis* (95). Similarly, the Hrc1-class T3SS of *Erwinia chrysanthemi* recognizes the secretion signals of the Ysc-class secreted proteins YopE and YopQ from *Yersinia*, and the Hrc1-class secreted avirulence proteins AvrB and AvrPto from *P. syringae* can be secreted by the Ysc-class T3SS of *Yersinia* (96). Such successful reciprocal secretion of proteins by different classes of T3SSs may suggest universal recognition of secretion signals. However, due to the lack of systematic studies and the fact that unsuccessful examples of heterologous secretion attempts are unlikely to be published, it remains unclear whether these examples are the exception or the rule. The discovery of effector- or effector-class-specific chaperones and translocation motifs suggests that effectors may not be universally recognized or translocated (97–99). Nevertheless, even if reciprocal secretion is not the rule, it provides a useful and straightforward strategy to test candidate effectors, as shown in this study.

The three candidate effectors from *X. ampelinus*, which were experimentally confirmed to contain a functional N-terminal type III secretion/translocation signal, share structural similarity with proteins of other plant-associated bacteria, including both pathogens (*Acidovorax* sp., *Pantoea agglomerans*, *P. amygdali*, *P. syringae*, *Ralstonia* sp., *Xanthomonas fragariae*), as well as symbionts (*Bradyrhizobium* sp.). Some of the proteins with similar structures are annotated as Ulp1 family isopeptidases or C48 family peptidases, known to be T3Es. Yet, there is no evidence that the *X. ampelinus* effectors have such a peptidase activity because the peptidase domains of these effectors do not align with the *X. ampelinus* amino acid sequence. The other homologs, which are annotated as hypothetical proteins, might also correspond to T3Es. Further studies need to determine how these effectors contribute to the infection of grapevine plants. Even more work is needed to understand the function of the other Hrp2/Hrc2-class T3SS, its candidate effectors, and co-regulated gene families, as exemplified by the *X. rhododendri* strain KACC 21265.

The direct or indirect detection of effectors by plant NBS-LRR (nucleotide-binding site leucine-rich repeat) immune receptors, also known as NLRs, activates a robust defense mechanism called effector-triggered immunity (100). Grapevine has a relatively high number of NLRs compared to other plant species (101). Some of these NLRs may recognize T3Es from *Xylophilus*, thus opening the door to strategies targeting type III effectors for resistance engineering and contributing to suitable control measures.

## Supplementary Material

Reviewer comments

## Data Availability

All Galaxy workflows are provided at https://doi.org/10.5281/zenodo.19733922.

## SUPPLEMENTAL MATERIAL

Text S1 spectrum.00500-26-s0001.pdf Legends for Data S1 to S3, Figures S1 to S3, and Tables S1 to S7.

Data S1 to S3 spectrum.00500-26-s0002.pdf Data S1: Multiple sequence alignment of SctV/FlhA homologs in *Xylophilus*. Data S2: HrpX-regulated protein families in *Xylophilus rhododendri*. Data S3: Multiple sequence alignments of novel T3Es (OG_1115, OG_1116, OG_3308).

Figure S1 to S3 spectrum.00500-26-s0003.pdf Fig. S1: Phylogenetic tree of representative SctU/FlhB proteins and homologs from *Xylophilus*. Fig. S2: Comparison of *Xylophilus flagellar* gene clusters. Fig. S3: Three-dimensional models of the tertiary structure of members of orthology groups OG_1115 and OG_1116*.*

Table S1 spectrum.00500-26-s0004.xlsx (a) Taxonomic classification of *Xylophilus* genome sequences using TYGS. (b) Taxonomic classification of *Xylophilus* genome sequences using MiGA.

Table S2 spectrum.00500-26-s0005.xlsx (a) Genetic relationships between *Xylophilus* genomes based on genome-wide average nucleotide identities. (b) Genetic relationships between *Xylophilus* genomes based on digital DNA-DNA hybridization.

Table S3 spectrum.00500-26-s0006.xlsx Predicted protein secretion systems in *Xylophilus* genomes using TXSScan.

Table S4 spectrum.00500-26-s0007.xlsx HrpX-regulated protein families in *Xylophilus rhododendri*.

Table S5 spectrum.00500-26-s0008.xlsx BLASTP hits of novel T3Es (OG_1115 and OG_1116).

Table S6 spectrum.00500-26-s0009.xlsx InterPro hits of representation homologs of novel T3Es (OG_1115 and OG_1116).

Table S7 spectrum.00500-26-s0010.xlsx FoldSeek hits of AlphaFold structural models of novel T3Es (OG_1115 and OG_1116).

OPEN PEER REVIEW reviewer-comments.pdf An accounting of the reviewer comments and feedback.

ASM does not own the copyrights to Supplemental Material that may be linked to, or accessed through, an article. The authors have granted ASM a non-exclusive, world-wide license to publish the Supplemental Material files. Please contact the corresponding author directly for reuse.
