# Supplementary material for: Identification of protein secretion systems and type III effectors in wood-associated bacteria of the genus Xylophilus
Source: Microbiol Spectr. 2026 Jul 6;14(8):e00500-26. doi: 10.1128/spectrum.00500-26 (PMC13436205; doi:10.1128/spectrum.00500-26)
Supplement: Supplementary file 1 [file reviewer-comments.pdf]

AMERICAN SOCIETY FOR MICROBIOLOGY | Microbiology Spectrum

Identification of protein secretion systems and type III effectors in wood-associated bacteria of the genus *Xylophilus*

Zoe Roux, Naama Wagner, Laurent Brottier, Tal Pupko, and Ralf Koebnik

Corresponding Author(s): Ralf Koebnik, Institut de Recherche pour le Développement

Review Timeline:

Submission Date:	February 12, 2026
Editorial Decision:	April 14, 2026
Revision Received:	May 6, 2026
Accepted:	June 8, 2026

Editor: Jing Han

Reviewer(s): Disclosure of reviewer identity is with reference to reviewer comments included in decision letter(s). The following individuals involved in review of your submission have agreed to reveal their identity: David J Studholme (Reviewer #1); Xiaofan Zhou (Reviewer #2)

Transaction Report:

(Note: With the exception of the correction of typographical or spelling errors that could be a source of ambiguity, letters and reports are not edited. The original formatting of letters and referee reports may not be reflected in this compilation.)

DOI: https://doi.org/10.1128/spectrum.00500-26

Re: Spectrum00500-26 (**Identification of protein secretion systems and type III effectors in wood-associated bacteria of the genus *Xylophilus***)

Dear Dr. Ralf Koebnik:

Thank you for the privilege of reviewing your work. Below you will find my comments, instructions from the Spectrum editorial office, and the reviewer comments.

Please return the manuscript within 60 days; if you cannot complete the modification within this time period, please contact me. If you do not wish to modify the manuscript and prefer to submit it to another journal, notify me immediately so that the manuscript may be formally withdrawn from consideration by Spectrum.

Revision Guidelines
To submit your modified manuscript, log into the submission site at https://spectrum.msubmit.net/cgi-bin/main.plex. Go to Author Tasks and click the appropriate manuscript title to begin. The information you entered when you first submitted the paper will be displayed; update this as necessary. Note the following requirements:

• Upload point-by-point responses to the issues raised by the reviewers in a file named "Response to Reviewers," NOT in your cover letter.
• Upload a compare copy of the manuscript (without figures) as a "Marked-Up Manuscript" file.
• Upload a clean .DOC/.DOCX version of the revised manuscript and remove the previous version.
• Each figure must be uploaded as a separate, editable, high-resolution file (TIFF or EPS preferred), and any multipanel figures must be assembled into one file.
• Any supplemental material intended for posting by ASM should be uploaded with their legends separate from the main manuscript. You can combine all supplemental material into one file (preferred) or split it into a maximum of 10 files with all associated legends included.

For complete guidelines on revision requirements, see our Submission and Review Process webpage. Submission of a paper that does not conform to guidelines may delay acceptance of your manuscript.

Data availability: ASM policy requires that data be available to the public upon online posting of the article, so please verify all links to sequence records, if present, and make sure that each number retrieves the full record of the data. If a new accession number is not linked or a link is broken, provide Spectrum production staff with the correct URL for the record. If the accession numbers for new data are not publicly accessible before the expected online posting of the article, publication may be delayed; please contact production staff (Spectrum@asmusa.org) immediately with the expected release date.

Publication Fees: For information on publication fees and which article types are subject to charges, visit our website. If your manuscript is accepted for publication and any fees apply, you will be contacted separately about payment during the production process; please follow the instructions in that e-mail. Arrangements for payment must be made before your article is published.

ASM Membership: Corresponding authors may join or renew ASM membership to obtain discounts on publication fees. Need to upgrade your membership level? Please contact Customer Service at Service@asmusa.org.

The ASM Journals program strives for constant improvement in our submission and publication process. Please tell us how we can improve your experience by taking this quick Author Survey.

Thank you for submitting your paper to Spectrum.

Sincerely,
Jing Han
Editor
Microbiology Spectrum

Reviewer #1 (Comments for the Author):

This manuscript makes an important contribution to the field by curating and surveying the available genome sequences for a largely neglected genus of plant pathogens. It identifies some interesting problems with the publicly available sequence data and discovers some interesting observations such as the discovery of new T3SS effectors, at least one of which (NBM50_02550) appears to be encoded within a bacteriophage. The work is of high quality and there are no major problems.

However, I have some suggestions for the authors to improve the work further.

(1) Page 1. Line 16. I would say "genome sequences" rather than "genomes".
(2) Page 1. Line 19. Delete the first occurrence of the word "were" so that the sentence makes sense.
(3) Page 1. Line 20. It is confusing to speak of "types of type". Would it be better to refer to "classes" or "sub-types" of T3SS? At line 11 on page 3.
(4) Page 1. Line 19. "one or two": is it one or is it two? Or perhaps it should be "one and two"?
(5) Page 1. Line 23. "predicted genes that are co-regulated": this is misleading. We do not actually know that those genes are co-regulated. Better to say something like "predicted which genes are co-regulated".
(6) Page 1. Line 28. "[an] isolate had ... many genes without homologs beyond the species". Please word this carefully to convey the intended meaning. The current wording seems to be simply saying that the rhododendron-associated species has many species-specific genes. But probably the authors meant to say something slightly different.
(7) Page 1. Lines 30-32. It is not obvious or self evident that the presence of T3SS effectors in a bacterium implies that effector-triggered immunity may exist in specific plants. Furthermore, I cannot see this same statement being made explicit in the Discussion section, though it is possibly implied on pages 24-25. If this statement is to remain in the Abstract, then please explain and justify it explicitly in the Discussion.
(8) Page 2. Line 25. What is the evidence to support this claim? Can you cite a reference? Is it the EPPO (2025) reference that gets cited later in the paragraph?
(9) Page 3. Lines 23-24. What's the evidence to support the claim about the most-conserved components? Can you cite a supporting reference or other data?
(10) Page 5. Line 7. Many readers will not be familiar with this plasmid. Can you briefly introduce it and/or cite a reference?
(11) Page 5. Lines 29-30. Please see https://tygs.dsmz.de/background/show for instructions on how to cite TYGS.
(12) Page 6 and elsewhere in the manuscript. The authors use Galaxy to perform several analyses. One of the great strengths of Galaxy is that it makes it easy to be transparent and open about methods. For example, it is possible to share and/or export the Galaxy workflow so that readers can see exactly what the researchers did, including software versions, parameter values and options selected, inputs and outputs etc. It is also possible to share Galaxy histories, though this might be less robust as the history may not persist in the long term if the user delete it etc. Please consider exporting and sharing the workflow from Galaxy and submitting as a supplementary file or to a repository such as Zenodo. That will allow others to repeat the analyses and ensure transparency.
(13) Page 6. Line 18. Strictly speaking, you should mention the version number for iTOL.
(14) Table 1. Indicate which strains are the type strains.
(15) Table 1. Given the possible confusion between Xylophilus and Xenophilus (!), it would be better to avoid abbreviating to "X.".
(16) Page 10. Lines 7 and 19. It is not really correct to say that these were classified as new species. Classified by whom? Where can we find descriptions of these supposed new species? It is more accurate to say that the strains do not fall within any currently described species.
(17) Line 10. It is confusing to talk about isolates originating from metagenome assemblies. How does one isolate bacteria from a metagenome assembly? Do they mean that the isolates were isolated from some microiome whose metagenome was sequenced? Or perhaps these are not isolates at all? Are they MAGs rather than sequences of isolates?
(18) Page 10. Line 15. This is an interesting observation. Consider also examining the genome assembly using ContEst16S to detect contamination.
(19) Page 10. Line 21. Flagged by whom?
(20) Page 10. Line 22. What criteria are used to flag a genome as "intermediate-quality"?
(21) Page 11. Line 2. Were these "genome sequences" actually MAGs?
(22) Page 11. Line 9. It would be better to delete "Xylophilus" from this line.
(23) Page 11. Line 13. How was this identity calculated? Is it the same as or different to ANI?
(24) Page 11. Line 18. What criteria are used to define significance here? Is this statistical significance?
(25) Page 12. Line 20. Please check whether sufficient methodological details have been provided so that a reader could exactly repeat this phylogenetic analysis.
(26) Page 13. Line 10. Briefly, how were these PIP boxes found? Was this using Effectidor?
(27) Page 13. Line 15. Just for information: the authors may be interested to learn that NCPPB 2930 was received as Xanthomonas, but sequencing revealed that it had been mis-identified.
(28) Page 16. Line 17. The DNA sequence is "located" not "encoded".
(29) Page 21. Line 24. It is good that the authors used InterPro to investigate their putative T3SS effectors. Please check whether InterProScan is mentioned in the Methods and reference(s) cited.
(30) Page 25. It would be interesting, at least for some readers, to comment on the taxonomic history of X. ampolinus. Why was it previously considered to be Xanthomonas and why was this changed (presumably because of phylogeny)? Does it share any phenotypic traits with Xanthomonas?

Reviewer #2 (Comments for the Author):

In the present study, Roux et al. conducted a comprehensive genome-wide identification of protein secretion systems,

specifically flagellar and Type III Secretion Systems (T3SS), within the genus Xylophilus. The authors first did a great job of clarifying the taxonomic status of the publicly available genome sequences, which provides a very reliable foundation for the subsequent evolutionary and functional analyses. Using computational predictions (such as Effectidor II), they identified a repertoire of candidate Type III effectors (T3Es). Furthermore, they successfully validated three novel effector candidates by employing an AvrBs1 reporter system in Xanthomonas campestris to confirm their functional secretion signals in host plants. Overall, the research logic is very clear, the methodology is robust, and the combination of in silico bioinformatic mining with in planta experimental validation makes this a very solid piece of work. It provides good theoretical significance for understanding the pathogenicity of X. ampelinus. However, there are a few areas where further clarification and minor corrections are needed to improve the quality of the manuscript before publication.

1. In the plant inoculation experiments, the authors utilized X. campestris 8004 as a surrogate system to test the secretion signals of the Xylophilus candidate effectors. While this cross-genus complementation works and is discussed appropriately, it would be helpful for the readers if the authors could add a brief sentence in the Discussion explaining why a homologous system (i.e., using X. ampelinus itself) was not utilized. Is it due to the lack of established genetic transformation protocols or suitable mutant strains for X. ampelinus?

2. In Tables 3 and 4, the authors list the candidate T3Es predicted by the Effectidor II algorithm. It is noticed that the scores range quite broadly (from 0.899 down to 0.251). Could the authors please explicitly state in the Methods section if a specific cut-off score was chosen to define a "high-confidence" candidate, and how this threshold was determined?

3. When discussing the AlphaFold3 structural modeling of the novel T3Es (OG_1115 and OG_1116), the authors note that the N-termini were largely predicted to be disordered with low confidence scores (pLDDT < 50). This is actually a very typical characteristic of T3SS effectors, as the N-terminal secretion signal must remain unfolded to pass through the narrow inner channel of the injectisome needle. It is highly recommended that the authors add a short discussion to link this "intrinsic disorder" structural prediction with the biological mechanism of Type III secretion, which will make the structural data more biologically meaningful.

Dear Editor,

Please find below our responses to the two anonymous reviewers. Our responses are shown in blue font.

Reviewer #1 (Comments for the Author):

This manuscript makes an important contribution to the field by curating and surveying the available genome sequences for a largely neglected genus of plant pathogens. It identifies some interesting problems with the publicly available sequence data and discovers some interesting observations such as the discovery of new T3SS effectors, at least one of which (NBM50_02550) appears to be encoded within a bacteriophage. The work is of high quality and there are no major problems. However, I have some suggestions for the authors to improve the work further.

We would like to thank the reviewer for this encouraging statement and the constructive feedback on our manuscript.

(1) Page 1. Line 16. I would say "genome sequences" rather than "genomes".

Thank you for this suggestion, which we have accepted.

(2) Page 1. Line 19. Delete the first occurrence of the word "were" so that the sentence makes sense.

We apologise for this confusing sentence, which arose from condensing the original, longer abstract to a maximum of 250 words.

(3) Page 1. Line 20. It is confusing to speak of "types of type". Would it be better to refer to "classes" or "sub-types" of T3SS? At line 11 on page 3.

We prefer to use the term ‘classes’ for the seven major groups of T3SSs (*i.e.*, SPI1, SPI2, Ysc, Hrc1, Hrc2, Rhiz, Chl). Since both ‘types’ belong to the Hrc2 class, we now call them ‘sub-classes’.

(4) Page 1. Line 19. "one or two": is it one or is it two? Or perhaps it should be "one and two"?

We appreciate the clarification and have accepted it.

(5) Page 1. Line 23. "predicted genes that are co-regulated": this is misleading. We do not actually know that those genes are co-regulated. Better to say something like "predicted which genes are co-regulated".

Thank you for this clarification. We have taken it into consideration.

(6) Page 1. Line 28. "[an] isolate had ... many genes without homologs beyond the species". Please word this carefully to convey the intended meaning. The current wording seems to be simply saying that the rhododendron-associated species has many

species-specific genes. But probably the authors meant to say something slightly different.

In fact, this is exactly what we meant to say. For many of these genes, *X. ampelinus* is the only BLAST hit. These genes are therefore indeed surprisingly restricted to a single species.

(7) Page 1. Lines 30-32. It is not obvious or self evident that the presence of T3SS effectors in a bacterium implies that effector-triggered immunity may exist in specific plants. Furthermore, I cannot see this same statement being made explicit in the Discussion section, though it is possibly implied on pages 24-25. If this statement is to remain in the Abstract, then please explain and justify it explicitly in the Discussion.

We added a short paragraph at the end of the Discussion to justify the statement in the Abstract:
"The direct or indirect detection of effectors by plant NBS-LRR (nucleotide-binding site leucine-rich repeat) immune receptors, also known as NLRs, activates a robust defence mechanism called effector-triggered immunity (Jones and Dangl 2006). Grapevine has a relatively high number of NLRs compared to other plant species (Li et al., 2023). Some of these NLRs may recognise T3Es from *Xylophilus*, thus opening the door to strategies targeting type III effectors for resistance engineering and contributing to suitable control measures."

(8) Page 2. Line 25. What is the evidence to support this claim? Can you cite a reference? Is it the EPPO (2025) reference that gets cited later in the paragraph?

This statement is derived from the "Scientific Opinion on the pest categorisation of *Xylophilus ampelinus*". We added the corresponding citation (EFSA Panel on Plant Health 2014).

(9) Page 3. Lines 23-24. What's the evidence to support the claim about the most-conserved components? Can you cite a supporting reference or other data?

Abby and Rocha (2012) calculated substitution rates per site of the eight T3SS core proteins and reconstructed the individual phylogenies, which supported a common descent of all non-flagellar T3SSs from a single ancestor (Figure S3 in their paper). This and other work demonstrated that SctT and SctV, along with the ATPase SctN, constitute the most conserved components of the T3SS. Since SctN is an ATPase with many homologs beyond the T3SS, this protein is less useful for identifying T3SSs. We slightly rephrased our statement and include a reference.

(10) Page 5. Line 7. Many readers will not be familiar with this plasmid. Can you briefly introduce it and/or cite a reference?

The citation of Kovach et al. (1995), which we provided a few lines later, has been moved to line 7.

(11) Page 5. Lines 29-30. Please see https://tygs.dsmz.de/background/show for instructions on how to cite TYGS.

Thank you for pointing out. We now cite the original reference for TYGS (Meier-Kolthoff and Göker 2019).

(12) Page 6 and elsewhere in the manuscript. The authors use Galaxy to perform several analyses. One of the great strengths of Galaxy is that it makes it easy to be transparent and open about methods. For example, it is possible to share and/or export the Galaxy workflow so that readers can see exactly what the researchers did, including software versions, parameter values and options selected, inputs and outputs etc. It is also possible to share Galaxy histories, though this might be less robust as the history may not persist in the long term if the user delete it etc. Please consider exporting and sharing the workflow from Galaxy and submitting as a supplementary file or to a repository such as Zenodo. That will allow others to repeat the analyses and ensure transparency.

All Galaxy workflows are provided at https://doi.org/10.5281/zenodo.19733922. This information was added to the end of the Material and Methods under 'Data availability'.

(13) Page 6. Line 18. Strictly speaking, you should mention the version number for iTOL.

We used iTOL version 6. This information was added.

(14) Table 1. Indicate which strains are the type strains.

Type strains are now indicated by a superscript T.

(15) Table 1. Given the possible confusion between *Xylophilus* and *Xenophilus* (!), it would be better to avoid abbreviating to "*X.*".

Done.

(16) Page 10. Lines 7 and 19. It is not really correct to say that these were classified as new species. Classified by whom? Where can we find descriptions of these supposed new species? It is more accurate to say that the strains do not fall within any currently described species.

That's correct. These are in fact proposals by the TYGS and MiGA servers. We have rephrased the statements accordingly.

(17) Line 10. It is confusing to talk about isolates originating from metagenome assemblies. How does one isolate bacteria from a metagenome assembly? Do they mean that the isolates were isolated from some microbiome whose metagenome was sequenced? Or perhaps these are not isolates at all? Are they MAGs rather than sequences of isolates?

We are sorry for this confusion, which we have accordingly rephrased.

(18) Page 10. Line 15. This is an interesting observation. Consider also examining the genome assembly using ContEst16S to detect contamination.

Thank you for this suggestion. Indeed, the ContEst16S algorithm also identified the *Xylophilus* Kf1 genome assembly as contaminated with DNA from *Staphylococcus epidermidis*. We added this information and the reference to the manuscript.

(19) Page 10. Line 21. Flagged by whom?

Flagged by TypeMat. We changed to "classified". See also next comment.

(20) Page 10. Line 22. What criteria are used to flag a genome as "intermediate-quality"?

The MiGA TypeMat quality report is based on estimates of the redundancy, completeness, and contamination, as described in Rodriguez-R et al., 2018. We added this detail.

(21) Page 11. Line 2. Were these "genome sequences" actually MAGs?

Yes, these are metagenome-assembled genome sequences. We clarified this detail.

(22) Page 11. Line 9. It would be better to delete "*Xylophilus*" from this line.

Done.

(23) Page 11. Line 13. How was this identity calculated? Is it the same as or different to ANI?

It is genome-wide ANI. We added this detail.

(24) Page 11. Line 18. What criteria are used to define significance here? Is this statistical significance?

The 70% cutoff comes from empirical observations in traditional DDH experiments where strains that clustered within the same species consistently showed ≥70% DNA reassociation. This threshold became the gold standard for prokaryotic species delineation. Indeed, the term "significantly" might not be appropriate and we replaced the term by "well".

(25) Page 12. Line 20. Please check whether sufficient methodological details have been provided so that a reader could exactly repeat this phylogenetic analysis.

We thank the reviewer for this comment. In the revised manuscript, we now explicitly state that default webserver parameters were used and clarify that phylogenetic trees were inferred with bootstrap support, using DSM 13620 as the outgroup.

(26) Page 13. Line 10. Briefly, how were these PIP boxes found? Was this using Effectidor?

No, this was not by Effectidor. It was a simple pattern scan in the Artemis genome browser, using TTCGB-N_{15}-TTCGB-$N_{30\text{-}32}$-YANNNT as a query. We added this detail in the Methods section under "Other bioinformatic tools".

(27) Page 13. Line 15. Just for information: the authors may be interested to learn that NCPPB 2930 was received as *Xanthomonas*, but sequencing revealed that it had been mis-identified.

Thanks for this interesting comment.

(28) Page 16. Line 17. The DNA sequence is "located" not "encoded".

Done.

(29) Page 21. Line 24. It is good that the authors used InterPro to investigate their putative T3SS effectors. Please check whether InterProScan is mentioned in the Methods and reference(s) cited.

Yes, InterProScan has been mentioned both in the Methods and in the references (Jones et al. 2014)

(30) Page 25. It would be interesting, at least for some readers, to comment on the taxonomic history of *X. ampelinus*. Why was it previously considered to be *Xanthomonas* and why was this changed (presumably because of phylogeny)? Does it share any phenotypic traits with *Xanthomonas*?

To address this point, we added the following paragraph:
"The taxonomic history of *X. ampelinus* reflects the broader evolution of bacterial systematics — from early reliance on phenotype and host association to modern genetic and phylogenetic methods. The pathogen causing bacterial necrosis of grapevine was formally described in 1969 by Panagopoulos as *Xanthomonas ampelina*, which was assigned to *Xanthomonas* largely because it looked and behaved like other xanthomonads, such as plant pathogenicity and slow growth at ambient temperatures, not because of evolutionary evidence. The bacterium was classified in the genus *Xanthomonas* as it is an aerobic, non-sporing, Gram negative rod with one polar flagellum, produced a yellow water-insoluble pigment and metabolized carbohydrates oxidatively (Panagopoulos 1969). Yet, the grapevine pathogen showed many significant physiological and biochemical differences in comparison with other described species of *Xanthomonas*. Using rRNA–DNA hybridization and supported by enzymatic characteristics, as analyzed using the API system, Willems et al. (1987b) later demonstrated that *X. ampelina* was not phylogenetically related to *Xanthomonas*. Its closest relatives were in Betaproteobacteria, not the Gammaproteobacteria where *Xanthomonas* belongs. Against this background, it is interesting to note that the effector repertoire of *Xylophilus ampelinus* bears a strong resemblance to the repertoires of other plant-pathogenic bacteria, including *Xanthomonas*."

References:

- Jones JD, Dangl JL (2006). The plant immune system. Nature 444: 323-329. doi: 10.1038/nature05286
- Li X, Ma L, Wang Y, Ye C, Guo C, Li Y, Mei X, Du F, Huang H (2023). PlantNLRatlas: a comprehensive dataset of full- and partial-length NLR resistance

genes across 100 chromosome-level plant genomes. Front. Plant Sci. 14: 1178069. doi: 10.3389/fpls.2023.1178069

- Panagopoulos CG (1969). The disease "Tsilik marasi" of grapevine: its description and identification of the causal agent (*Xanthomonas ampelina* sp. nov.). Ann. Inst. Phytopathol. Benaki 9: 59-81.

Reviewer #2 (Comments for the Author):

In the present study, Roux et al. conducted a comprehensive genome-wide identification of protein secretion systems, specifically flagellar and Type III Secretion Systems (T3SS), within the genus *Xylophilus*. The authors first did a great job of clarifying the taxonomic status of the publicly available genome sequences, which provides a very reliable foundation for the subsequent evolutionary and functional analyses. Using computational predictions (such as Effectidor II), they identified a repertoire of candidate Type III effectors (T3Es). Furthermore, they successfully validated three novel effector candidates by employing an AvrBs1 reporter system in Xanthomonas campestris to confirm their functional secretion signals in host plants. Overall, the research logic is very clear, the methodology is robust, and the combination of in silico bioinformatic mining with in planta experimental validation makes this a very solid piece of work. It provides good theoretical significance for understanding the pathogenicity of *X. ampelinus*. However, there are a few areas where further clarification and minor corrections are needed to improve the quality of the manuscript before publication.

We appreciate the positive and constructive feedback from this reviewer.

1. In the plant inoculation experiments, the authors utilized *X. campestris* 8004 as a surrogate system to test the secretion signals of the *Xylophilus* candidate effectors. While this cross-genus complementation works and is discussed appropriately, it would be helpful for the readers if the authors could add a brief sentence in the Discussion explaining why a homologous system (i.e., using *X. ampelinus* itself) was not utilized. Is it due to the lack of established genetic transformation protocols or suitable mutant strains for *X. ampelinus*?

The genus *Xylophilus* is poorly studied genetically, with limited genomic resources and little development of molecular tools. There are no standard cloning vectors, shuttle plasmids, or expression systems reported as being optimized for *Xylophilus*. It would have been necessary to adapt broad-host-range vectors, and empirically test transformation/conjugation conditions as there's no standardized protocol available. Strictly speaking, a homologous system would involve using an avirulence gene from *Xylophilus* rather than *Xanthomonas*. However, even if a homologue of *avrBs1* was identified in *X. ampelinus*, it is unclear whether this gene would trigger a hypersensitive response in ECW-10R pepper plants. We also do not know whether *X. ampelinus* would trigger a hypersensitive response independently of the *avrBs1* homologue. If so, either another plant would need to be found for the inoculation experiments or a mutant of *Xylophilus* would need to be created that does not trigger an *avrBs1*-independent hypersensitive response. Working with grapevine plants would not be any easier here, as no gene-for-gene resistance mechanism has been described for this pathosystem. Taking all this into account, *X. campestris* is undoubtedly the better option, even if the

Xanthomonas T3SS might be unable to efficiently transport all effectors from *Xylophilus*. We have added a brief explanation of why we used a surrogate system:

"Rather than using *X. ampelinus* itself, we used *X. campestris* as a surrogate system to test the secretion signals of *Xylophilus* candidate effectors. This choice was due to the absence of suitable reporter plasmids, hosts with corresponding resistance genes, mutants in *Xylophilus*, as well as the lack of established genetic transformation protocols. Interestingly, the surrogate system proved effective in studying effector candidates from other taxa."

2. In Tables 3 and 4, the authors list the candidate T3Es predicted by the Effectidor II algorithm. It is noticed that the scores range quite broadly (from 0.899 down to 0.251). Could the authors please explicitly state in the Methods section if a specific cut-off score was chosen to define a "high-confidence" candidate, and how this threshold was determined?

We agree that the range of prediction scores is broad, reflecting variation in how strongly candidate proteins share features with known type III effectors. To define a consistent set of candidates, we included all predictions with scores >0.25 in Tables 3 and 4. This cutoff was chosen empirically: candidates with scores close to this threshold still showed sequence similarity to known effectors (e.g., OG_1722 in Table 3, score = 0.256, similar to RipW; and OG_7234 in Table 4, score = 0.251, similar to RipL), whereas no such similarities were observed for predictions below this value. Following the reviewer's suggestion, we have now clarified this point in the Methods section. Specifically, we added the following sentence to the paragraph beginning "To predict T3Es, we employed Effectidor II…":
"We included all predictions with scores higher than 0.25. This cutoff was selected because candidates with scores near this threshold showed sequence similarity to known effectors, whereas candidates below this threshold did not."

3. When discussing the AlphaFold3 structural modeling of the novel T3Es (OG_1115 and OG_1116), the authors note that the N-termini were largely predicted to be disordered with low confidence scores (pLDDT < 50). This is actually a very typical characteristic of T3SS effectors, as the N-terminal secretion signal must remain unfolded to pass through the narrow inner channel of the injectisome needle. It is highly recommended that the authors add a short discussion to link this "intrinsic disorder" structural prediction with the biological mechanism of Type III secretion, which will make the structural data more biologically meaningful.

Indeed, previous studies have shown that many T3Es possess intrinsically disordered N-terminal regions, consistent with our findings, and have proposed a model explaining how this feature may facilitate translocation. In response to this comment, we revised the relevant paragraph as follows:

In the manuscript, we modified the relevant paragraph to include this information:
"Notably, the N-termini were largely predicted to be disordered…slightly to 0.42 and 0.40, respectively. This is consistent with previous studies showing that many T3Es possess intrinsically disordered N-terminal regions. For example, Gazi et al. (2009) reported that 75% of *Pseudomonas syringae* pv. *tomato* T3Es exhibit a significant propensity for structural disorder within their first 50 N-terminal residues. In addition,

LeBlanc et al. (2021) showed that efficient translocation requires T3Es to be mechanically labile. Their findings support a model in which unfolding and secretion initiate at the N-terminus, with the intrinsic disorder of effector N-terminal regions likely facilitating this process by reducing the energetic barrier to unfolding initiation.

References:

- Gazi AD, Charova SN, Panopoulos NJ, Kokkinidis M (2009). Coiled-coils in type III secretion systems: structural flexibility, disorder and biological implications. Cell. Microbiol. 11: 719-729. doi: 10.1111/j.1462-5822.2009.01297.x
- LeBlanc M, Fink MR, Perkins TT, Sousa MC (2021). Type III secretion system effector proteins are mechanically labile. Proc. Natl. Acad. Sci. U.S.A. 118: e2019566118. doi: 10.1073/pnas.2019566118

Re: Spectrum00500-26R1 (**Identification of protein secretion systems and type III effectors in wood-associated bacteria of the genus *Xylophilus***)

Dear Dr. Ralf Koebnik:

Your manuscript has been accepted, and I am forwarding it to the ASM production staff for publication. Your paper will first be checked to make sure all elements meet the technical requirements. ASM staff will contact you if anything needs to be revised before copyediting and production can begin. Otherwise, you will be notified when your proofs are ready to be viewed.

Data Availability: ASM policy requires that data be available to the public upon online posting of the article, so please verify all links to sequence records, if present, and make sure that each number retrieves the full record of the data. If a new accession number is not linked or a link is broken, provide production staff with the correct URL for the record. If the accession numbers for new data are not publicly accessible before the expected online posting of the article, publication may be delayed; please contact ASM production staff immediately with the expected release date.

Publication Fees: For information on publication fees and which article types have charges, please visit our website. We have partnered with Copyright Clearance Center (CCC) to collect author charges. If fees apply to your paper, you will receive a message from no-reply@copyright.com with further instructions. For questions related to paying charges through RightsLink, please contact CCC at ASM_Support@copyright.com or toll free at +1-877-622-5543. CCC makes every attempt to respond to all emails within 24 hours.

ASM Membership: Corresponding authors may join or renew ASM membership to obtain discounts on publication fees. Need to upgrade your membership level? Please contact Customer Service at Service@asmusa.org.

Embargo Policy: A press release may be issued as soon as the manuscript is posted on the Spectrum Latest Articles webpage. The corresponding author will receive an email with the subject line "ASM Journals Author Services Notification" when the article is available online.

The ASM Journals program strives for constant improvement in our submission and publication process. Please tell us how we can improve your experience by taking this quick Author Survey.

Thank you for submitting your paper to Spectrum.

Sincerely,
Jing Han
Editor
Microbiology Spectrum
