# Supplementary material for: Identification of protein secretion systems and type III effectors in wood-associated bacteria of the genus Xylophilus
Source: Microbiol Spectr. 2026 Jul 6;14(8):e00500-26. doi: 10.1128/spectrum.00500-26 (PMC13436205; doi:10.1128/spectrum.00500-26)
Supplement: Supplementary file 2 [file spectrum.00500-26-s0001.pdf]

Identification of protein secretion systems and type III effectors in wood-associated bacteria of the genus *Xylophilus*

Zoe Roux[1], Naama Wagner[2], Laurent Brottier[1], Tal Pupko[2], and Ralf Koebnik[1,*]

[1] PHIM, Université de Montpellier, Cirad, INRAE, Institut Agro, IRD, Montpellier, France

[2] Shmunis School of Biomedicine and Cancer Research, George S. Wise Faculty of Life Sciences, Tel Aviv University, Tel-Aviv, Israel

**Author for Correspondence*

Legends to the supplemental data, figures and tables

Data S1. Multiple sequence alignment of SctV/FlhA homologs in *Xylophilus*.

Data S2. HrpX-regulated protein families in *Xylophilus rhododendri*.

Data S3. Multiple sequence alignments of novel T3Es (OG_1115, OG_1116, OG_3308).

Figure S1. Phylogenetic tree of representative SctU/FlhB proteins and homologs from *Xylophilus*. *Xylophilus* sequences are boxed with the three flagellar proteins in yellow (type 1), orange (type 2) and red (type 3), and the Sct proteins in green (T3SS#1) and blue (T3SS#2). Strain CFBP 1192 was omitted because it is a clone of strain CECT7646. Sequences that were used to identify SctV/FlhA homologs in proteomes of *Xylophilus* are indicated by a blue dot.

Figure S2. Comparison of *Xylophilus* flagellar gene clusters. Corresponding regions, extracted from GenBank files, were compared and visualized with Clinker. Three types of flagellar gene clusters were observed, as supported by synteny and sequence similarities. Flagellar genes are indicated as follows: flagellin genes in yellow, *flg* genes in red, *flh* genes in purple, *fli* genes in blue, *mot* genes in green and *che* genes in orange.

Figure S3. Three-dimensional models of the tertiary structure of members of orthology groups OG_1115 and OG_1116. Snapshots of the predicted structure, colored according to the pIDDT confidence scores, and the expected position errors are shown.

Table S1. (a) Taxonomic classification of *Xylophilus* genome sequences using TYGS. (b) Taxonomic classification of *Xylophilus* genome sequences using MiGA.

Table S2. (a) Genetic relationships between *Xylophilus* genomes based on genome-wide average nucleotide identities. (b) Genetic relationships between *Xylophilus* genomes based on digital DNA-DNA hybridization.

Table S3. Predicted protein secretion systems in *Xylophilus* genomes using TXSScan.

Table S4. HrpX-regulated protein families in *Xylophilus rhododendri*.

Table S5. BLASTP hits of novel T3Es (OG_1115 and OG_1116).

Table S6. InterPro hits of representation homologs of novel T3Es (OG_1115 and OG_1116).

Table S7. FoldSeek hits of AlphaFold structural models of novel T3Es (OG_1115 and OG_1116).
