# Supplementary material for: Identification of protein secretion systems and type III effectors in wood-associated bacteria of the genus Xylophilus
Source: Microbiol Spectr. 2026 Jul 6;14(8):e00500-26. doi: 10.1128/spectrum.00500-26 (PMC13436205; doi:10.1128/spectrum.00500-26)
Supplement: Supplementary file 4 [file spectrum.00500-26-s0003.pdf]

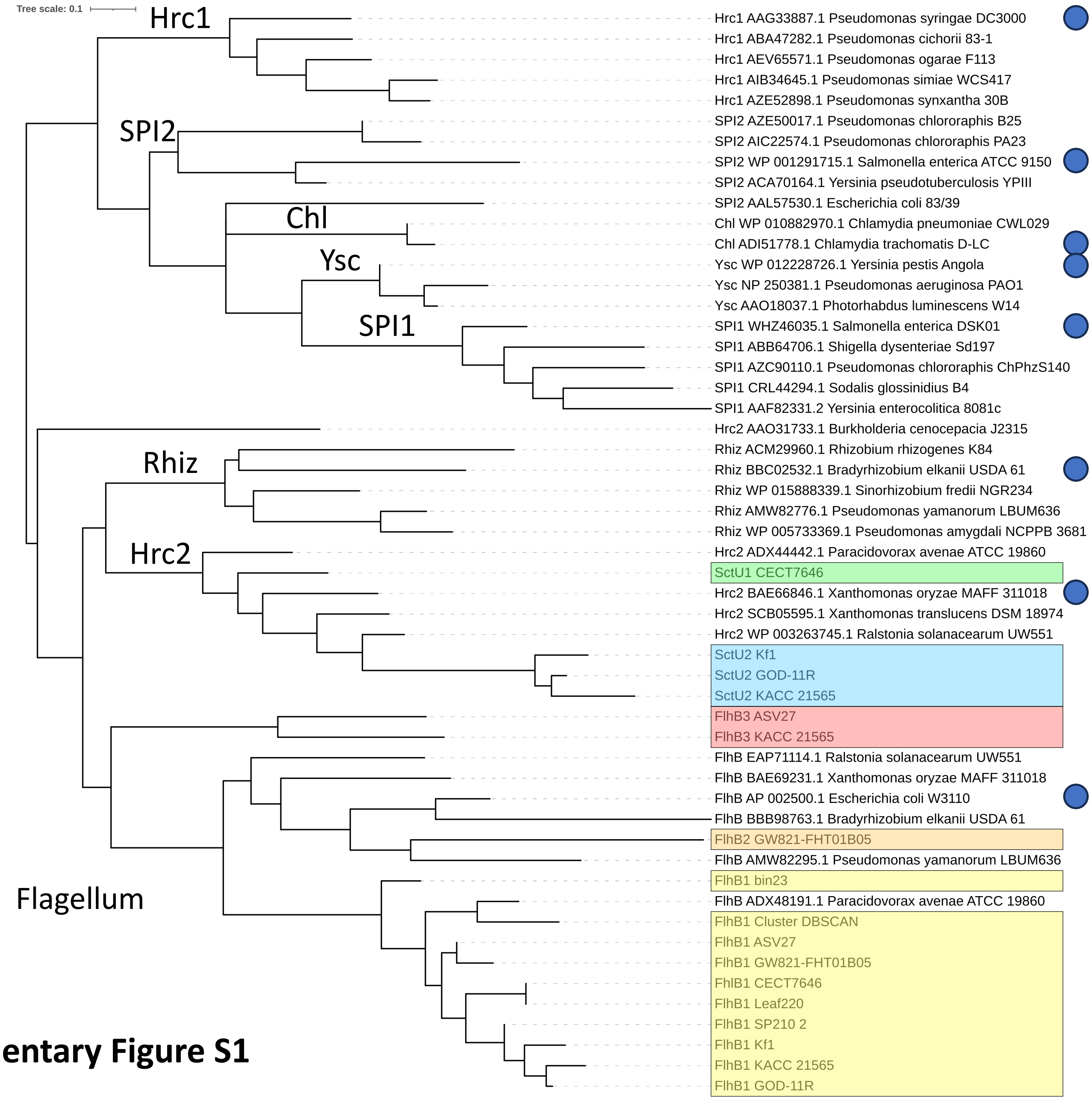

Supplementary Figure S1

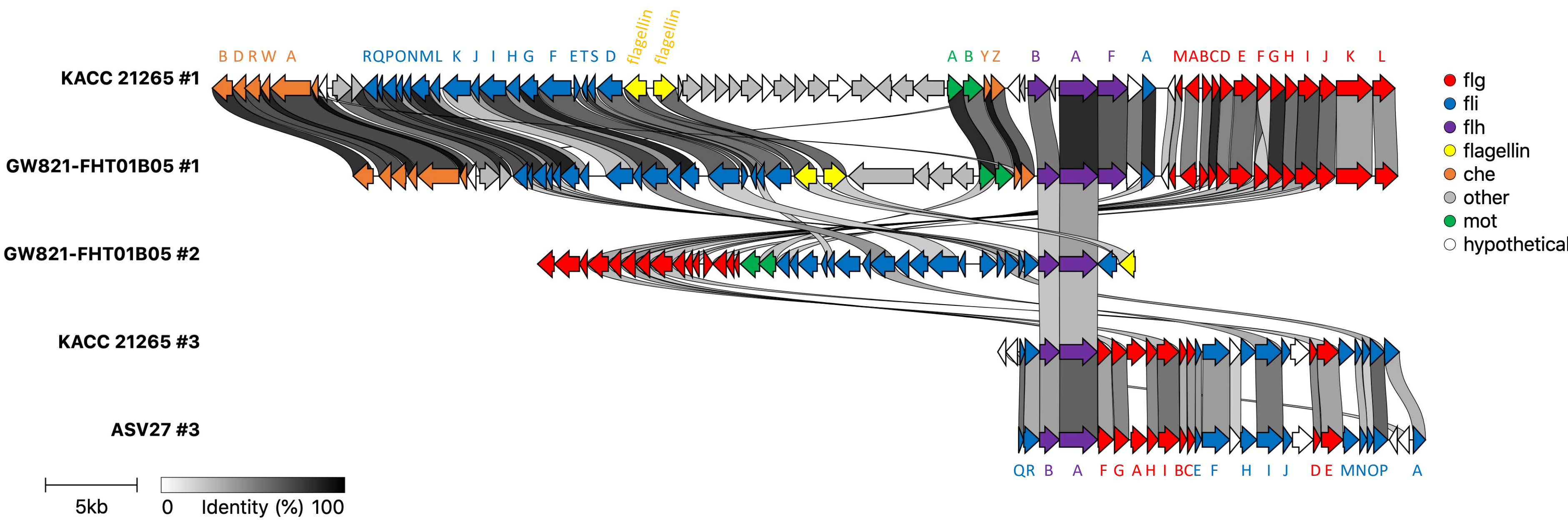

Supplementary Figure S2

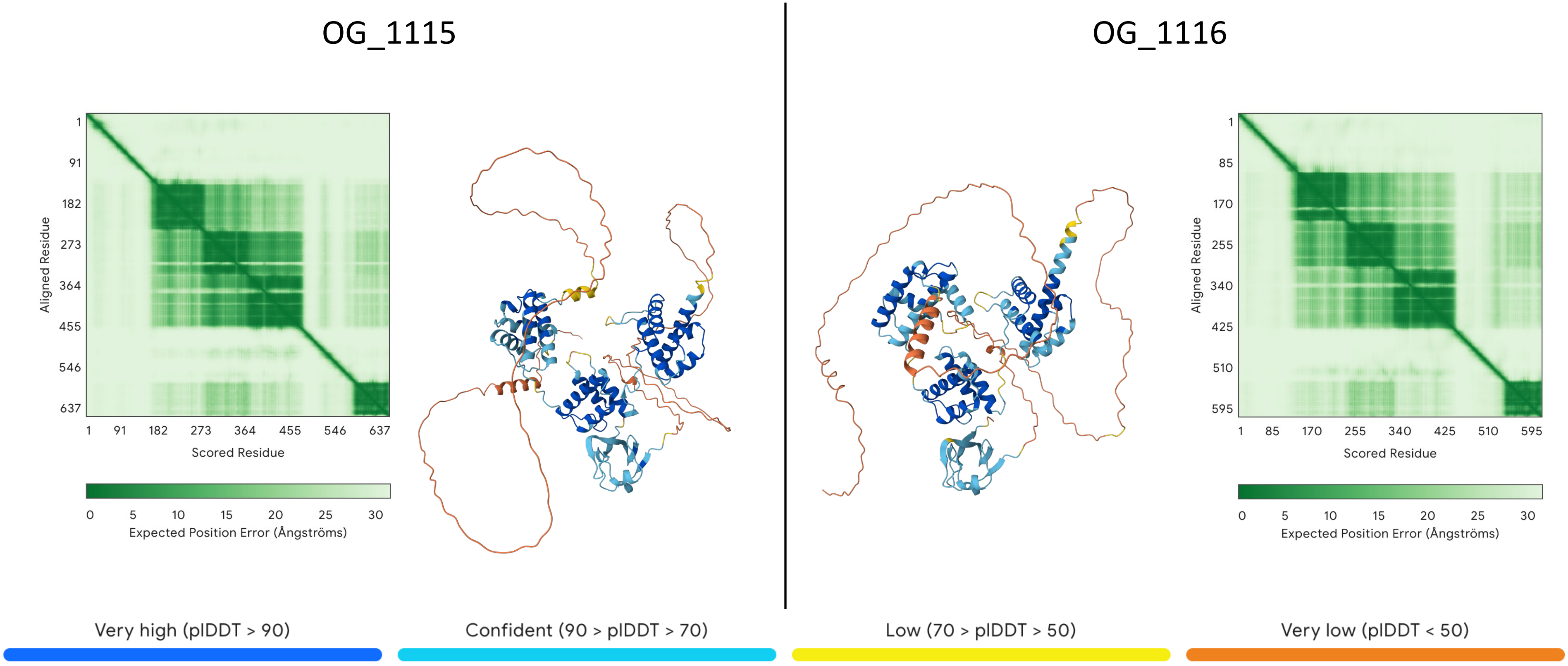

Supplementary Figure S3
